# Novel 4-Methylumbelliferone Amide Derivatives: Synthesis, Characterization and Pesticidal Activities

**DOI:** 10.3390/molecules23010122

**Published:** 2018-01-08

**Authors:** Yan Wei, Kai-Long Miao, Shuang-Hong Hao

**Affiliations:** Research Center of Agro-Bionic Engineering & Technology of Shandong Province, College of Chemistry & Pharm., Qingdao Agricultural University, Qingdao 266109, China; haoyihe@sohu.com (Y.W.); m18663915715@163.com (K.-L.M.)

**Keywords:** 4-methylumbelliferone, synthesis, acaricidal activity, herbicidal activity, antifungal activity

## Abstract

A series of novel 4-methylumbelliferone amide derivatives were designed, synthesized and characterized by ^1^H NMR, ^13^C NMR and HR-ESI-MS. The structures of compounds **4bd** and **4be** (compounds named by authors) were further confirmed by X-ray single crystal diffraction. The acaricidal, herbicidal and antifungal activities of the synthesized compounds were assayed for their potential use as pesticide. The results indicated that compounds **4bi**, **4ac** and **4bd** were strong acaricidals against *Tetranychus cinnabarinus*, with 72h corrected mortalities of greater than 80% at 1000 mg/L. Meanwhile, compounds **4bh** and **4bf** exhibit the strongest inhibition against the taproot development of *Digitaria sanguinalis* and *Chenopodium glaucum*, and were even more potent than the commercial herbicide Acetochlor against *D. sanguinalis*. In addition, compounds **4bk**, **4bh** and **4bp** showed the highest antifungal activity against the mycelium growth of *Valsa mali*, which makes them more effective than commercial fungicide Carbendazim.

## 1. Introduction

The coumarin scaffold is an important structure type which exists in a wide class of natural and synthetic compounds [1]. Derivatives of coumarin have broad applications in the fields of perfume, food additives, cosmetics, optical brighteners [2] and dye lasers [3]. They also exhibit many bioactivities of the following categories: anticoagulant [4], vasodilator [5], sedation and hypnosis [6], analgesic and hypothermy [7], estrogenic [8], antioxidant [9], dermal photosensitization [10], antibacterial [11], antifungal [12], moluscicidal and anthelmintic [13]. Among the various coumarins, 4-methylumbelliferone (7-hydroxy-4-methylcoumarin, hymecromone) and its derivatives have been used as fluorescent probes to detect Hg^2+^ in neat aqueous solutions [14], and hypochlorite in tap water and cancer cells [15], as well as to assay lipases and esterases [16]. 4-Methylumbelliferone derivatives also possess diverse biological properties, such as antipsychotic [17], antidepressant [18], anaphylaxis [19], hypoglycemic [20], antioxidant [21], anti-inflammatory [22], antitumor [23,24], antibacterial [25] and antifungal [26] properties. Even though some coumarins are toxic, 4-methylumbelliferone is a safe compound used as active ingredient in several approved drugs [27,28].

4-Methylumbelliferone and its derivatives exhibited interesting pesticidal activities as well. These compounds displayed strong inhibition to weeds *Setaria viridis* and *Amaranthus retroflexus*. The C7 hydroxyl group was considered as a potentially active site and a methyl substitution at the C4 position contributed significantly to the activity [29]. 4-Methylumbelliferone derivatives also showed strong growth inhibition against phytopathogenic fungi *Alternaria alternata*, *Alternaria solani*, *Botrytis cinerea* and *Fusarium oxysporum*, and a C4 methyl in the compounds contributed to the fungicidal activity [30]. Moreover, brominated 4-methylumbelliferone showed remarkable larvicidal and ovicidal activities against vectors *Aedes aegypti* and *Culex quinquefasciatus* [31]. 4-Methylumbelliferone esters of the chrysanthemic acid type could be metabolized by glutathione S-transferase from the mosquito *Culex pipiens pipiens* [32]. The Schiff base and its metal complexes of 4-methylumbelliferone derivatives showed anthelmintic to *Pheretima posthuma* [33].

The amide group is a common functional group in natural compounds. Many commercial pesticidal compounds have acylamino group in the molecule, for example carbamates and benzoylphenyl urea insecticides, anilide fungicides, ureas, amides and carbamates herbicides [34]. As a continuous study on the development of novel pesticides based on the coumarin scaffold [35,36], by introducing amides to 4-methylumbelliferone, a series of novel 4-methylumbelliferone amide derivatives were designed and synthesized through the principle of bioactive substructure combination. The acaricidal, herbicidal and antifungal activities of these new compounds were tested.

## 2. Results and Discussion

### 2.1. Chemistry

**Scheme 1:**
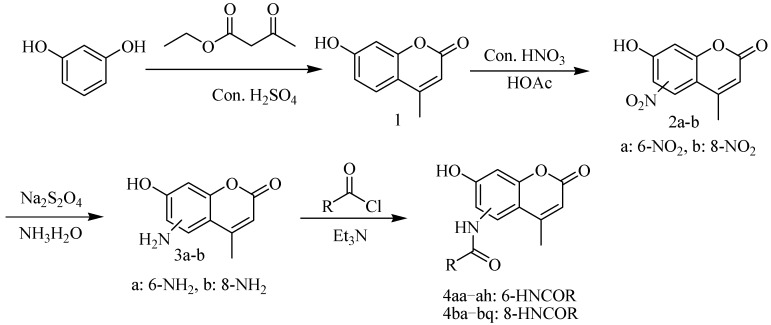
Synthetic route of target compounds.

**Figure 1:**
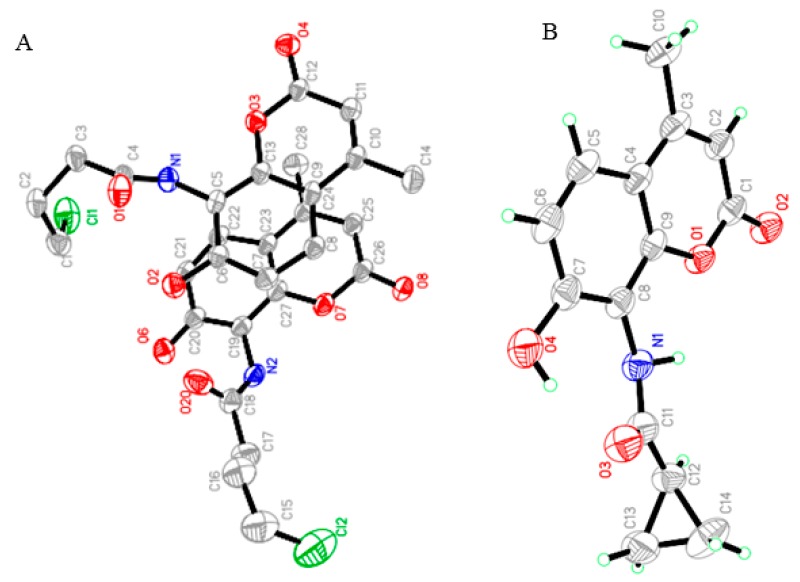
X-ray crystal structure of compounds **4bd** (**A**) and **4be** (**B**).

The synthetic route of the target compounds is illustrated in Scheme 1. Resorcinol and ethylacetoacetate occurred following a Pechmann reaction with catalyst of Con. H_2_SO_4_ to produce compound **1** [22]. Nitration of 4-methylumbelliferone can be achieved with HNO_3_/H_2_SO_4_ [37], HNO_3_/HOAc [37], Cr(NO_3_)_3_/Ac_2_O [38], (NH_2_)_2_CNH·HNO_3_/H_2_SO_4_ [39], Ce(NH_4_)_2_(NO_3_)_6_/H_2_O_2_ [40] and NO_2_BF_4_ [41]. Each method was reported to have some regioselectivity, but it is obvious that HNO_3_/HOAc was more safe and accessible. Thus, in the present study, compound **1** was nitrated by Conc. HNO_3_ in acetic acid to give the mixture of compounds **2a** and **2b** [37]. Nitro 4-methylumbelliferone can be reduced by SnCl_2_/HCl [22], Na_2_S_2_O_4_/NH_3_·H_2_O [42] and d-glucose/KOH [43] to give an amino product. Considering the temperature requirement, time requirements and ease of the operation, Na_2_S_2_O_4_/NH_3_·H_2_O was used as reductant. Thus the mixtures **2a** and **2b** were reduced in whole with Na_2_S_2_O_4_ in aqueous NH_3_ to obtain the mixture of compounds **3a** and **3b** [41]. The target compounds **4aa**–**4ah** and **4ba**–**4bq** were furnished by the acylazation of compounds **3a** and **3b**, respectively, with a series of acyl chlorides catalyzed by triethylamine [44]. The structures of all target compounds were well characterized by ^1^H NMR, ^13^C NMR and HR-ESI-MS. Additionally, to confirm the three-dimensional structural information of the target compounds, the single-crystal structures of **4bd** and **4be** were determined by X-ray crystallography as illustrated in Figure 1. Crystallographic data (excluding structure factors) for the structures of **4bd** and **4be** have been deposited with the Cambridge Crystallographic Data Centre as supplementary publication CCDC 1584028 and 1584029 (12 Union Road, Cambridge CB2 1EZ, UK; Fax: +44 1223 336033; E-mail: deposit@ccdc.cam.ac.uk). The C6 substituted target compounds were more polar than the C8 substituted compounds with the same group. Many fewer pure C6 substituted target compounds were created by column chromatography (CC). Moreover the C6 substituted target compounds **4aa**–**4ah** were dissolved by DMSO-*d*_6_ to determine the NMR, while the C8 substituted compounds **4ba**–**4bq** were dissolved by CDCl_3_ for NMR.

### 2.2. Acaricidal Activities

The acaricidal activities of the 25 synthesized target compounds against the phytophagous mite *T. cinnabarinus* were evaluated. The 72 h corrected mortalities of the mitetreated bythe compounds are listed in Table 1. The results indicate that most of the title compounds exhibited moderate to high acaricidal potency. Among them, the most attractive compounds were **4bi**, **4ac** and **4bd** with 72 h corrected mortalities of greater than 80% at 1000 mg/L. Especially **4bi** showed equal toxicity with the commercial acaricide Bifenazate. In the 6-substituted derivatives, the methacryloyl substituted compound **4ac** was the most active, followed by the aroyl substituted compounds **4ae**–**4ah**. The alkanoyl substituted compounds **4aa**, **4ab** and **4ad** were inferior. Meanwhile, in the 8-substituted derivatives, the most active was the hydrocinnamoyl substituted compound **4bi**, though its 6-position isomer **4ad** and cinnyl substituted compound **4bj** were much less potent. In the chain alkanoyl substituted compounds **4ba**–**4bc**, long chains seem favorable for the activity, however the 4-chloro-butanoyl substituted compound **4bd** was an exception. The smaller cyclopropyl formyl substituted compound was more active than the larger cyclohexyl formyl substituted one. The electron withdrawing groups on the aromatic ring of the amide group were favorable for the activity, but the 4-methyl benzoyl substituted compound **4bk** was more active.

### 2.3. Herbicidal Activities

The herbicidal activities of the target compounds against the taproot and caulis development of dicotyledonous weed *C. glaucum* and monocotyledonous weed *D. sanguinalis* were screened. The inhibitory rates of the compounds an effectiveness greater than 30% at 100 mg/L to at least one organ of the weeds are displayed in Table 2. The data indicate that more compounds show stronger inhibition against *D. sanguinalis* than against *C. glaucum*. More specifically, compounds **4bh** and **4bf** exhibit the strongest inhibition against the taproot development of *D. sanguinalis*, which makes them even more potent than the commercial herbicide Acetochlor. In fact **4bh** and **4bf** were the most potent against the taproot development of *C. glaucum*. Regarding effectiveness against *D. sanguinalis*, the chain alkanoylsubstituted compounds **4ba**–**4bd** (long chain compounds) seem unfavorable for the activity. Meanwhile the larger cyclohexyl formyl substituted compound was more active than the smaller cyclopropyl formyl substituted one.

### 2.4. Antifungal Activities

The in vitro antifungal activities of the target compounds against the mycelium growth of the phytopathogens *Colletotrichum glecosporioides*, *B. cinerea*, *F. oxysporum* and *V. mali* were assayed. The inhibitory rates of the compounds with effectiveness greater than 30% at 100 mg/L are listed in Table 3. The data indicate that more compounds showed stronger inhibition against *V. mali* than against the other 3 plant disease fungi. More specifically, compounds **4bk**, **4bh** and **4bp** exhibited the strongest inhibition against the mycelium growth of *V. mali*, which makes them even more potent than the commercial fungicide Carbendazim. Furthermore, the effectiveness of compounds **4be**, **4bi** and **4bd** against *B. cinerea* were superior than or comparable with that of the fungicide Carbendazim, though they are all only moderately active against the fungi. The inhibition of compound **4bq** against *F. oxysporium* was comparable with that of fungicide Carbendazim.

## 3. Experimental Section

### 3.1. Chemistry

All chemicals were obtained from commercial sources and used without further purification. Analytical thin-layer chromatography (TLC) was performed with silica gel plates using silica gel 60 GF_254_ (Qingdao Haiyang Chemical Co., Ltd., Qingdao, China). Melting points were determined on a WRS-1B digital melting-point apparatus (Shanghai Precision Optical Instrument Co., Ltd., Shanghai, China) without further calibration. Nuclear magnetic resonance spectra (NMR) were recorded on a Bruker Avance III HD 500 MHz instrument (Bruker, Faellanden, Switzerland) in CDCl_3_ or DMSO-*d*_6_ (^1^H at 500 MHz and ^13^C at 126 MHz) using tetramethylsilane (TMS) as the internal standard. High-resolution mass spectra (HRMS) were carried out with an IonSpec 4.7 T FTMS instrument. Single-crystal structure was determined by a Bruker AXS D8 QUEST X-ray single crystal diffractometer.

#### 3.1.1. Synthesis of **1**

A solution of resorcinol (5.5 g, 50 mmol) and ethyl acetoacetate (6.5 g, 50 mmol) in ethanol (10 mL) was added dropwise to H_2_SO_4_ (5 mL) with stirring at 0–5 °C. After the complete addition, the reaction mixture was stirred for 4 h at room temperature. Then the reaction mixture was poured onto ice-water (100 mL) with vigorous stirring for 1h. The white precipitate which formed was collected, and washed with cold water to neutral. The product was dried and crystallized, and separated from ethanol. Yield 88%, m.p. 185–186 °C (as reported) [22].

#### 3.1.2. Synthesis of **2a** and **2b**

To a solution of **1** (8.8 g, 50 mmol) in acetic acid (20 mL) kept at a temperature below 10 °C, a mixture of 65% nitric acid (5.8 g, 60 mmol) and acetic acid (20 mL) was added dropwise with stirring. After the complete addition, the reaction mixture was stirred for another 6 h at room temperature and then poured onto ice-water. The orange precipitate obtained was filtered off, washed with water to neutral, and air-dried. The solid product was washed with hot acetonitrile to give the yellow mixtures **2a** and **2b** (3:1) with a yield of 70% [37].

#### 3.1.3. Synthesis of **3a** and **3b**

To a suspension of **2a** and **2b** mixtures (11.1 g, 50 mmol) in 60 mL of concentrated aqueous ammonia, 70 mL of 15% sodium hydrosulfite was added slowly with stirring at room temperature. After the complete addition, the reaction mixture was stirred for 6 h until the color changed from bright orange to light green. The mixture was then boiled for 15 min, cooled to 5 °C, and the solid was filtered off to give the mixtures **3a** and **3b** (3:1) with a yield of 93% [41].

#### 3.1.4. General Procedure for Synthesis of 4-Methylumbelliferone Amide Derivatives (**4aa**–**4ah**, **4ba**–**4bq**)

To a suspension of 1.91 g of 6-amino-7-hydroxyl-4-methylcourmin (10 mmol) and 100 uL of triethylamine in 30 mL of dichloromethane (DCM), a solution of acyl chloride (10 mmol) in 20 mL DCM was added with stirring at 0–5 °C. The mixture was allowed to restore to room temperature and was stirred until the end of the reaction. The resulting organic mixture was washed with 30 mL of water (producing Na_2_SO_4_), then dried, filtered and concentrated in vacuo sequentially. The obtained residue was purified by silica gel column chromatography (petroleum ether–ethyl acetate = 1:1 for **4aa**–**4ah**, petroleum ether–ethyl acetate = 4:1 for **4ba**–**4bq**) to give the target compounds [44]. The yield, appearance, m.p., the ^1^H-NMR and ^13^C NMR spectra of Compounds **4aa**–**4bq** as Supplementary Materials, NMR and HRMS dates of the synthesized compounds are listed below.

*6-(n-Butanoylamino)-4-methylumbelliferone* (**4aa**). Pale yellow solid; yield: 71.2%; m.p. 251.6–254.8 °C; ^1^H NMR δ: 11.10 (s, 1H), 9.27 (s, 1H), 8.24 (s, 1H), 6.83 (s, 1H), 6.18 (s, 1H), 2.40 (t, *J* = 7.0 Hz, 2H), 2.35 (s, 3H), 1.67–1.58 (m, 2H), 0.93 (t, *J* = 7.5 Hz, 3H); ^13^C NMR δ: 172.29, 160.77, 153.87, 152.55, 151.05, 124.51, 118.06, 111.81, 111.26, 102.66, 38.29, 19.09, 18.66, 14.13; HR-ESI-MS *m*/*z*: 260.0918 [M − H]^−^ (calculated for C_14_H_14_NO_4_, 260.0923).

*6-(Cyclohexyl-formylamino)-4-methylumbelliferone* (**4ab**). Yellow solid; yield: 56.3%; m.p. 116.5–117.8 °C; ^1^H NMR δ: 11.08 (s, 1H), 9.15 (s, 1H), 8.25 (s, 1H), 6.81 (s, 1H), 6.16 (s, 1H), 2.58–2.53 (m, 1H), 2.34 (s, 3H), 1.82–1.73 (m, 3H), 1.66–1.63 (m, 1H), 1.44 (qd, *J*_1_ = 12 Hz, *J*_2_ = 2.5 Hz, 2H), 1.32–1.13 (m, 4H); ^13^C NMR δ: 175.37, 160.74, 153.85, 152.39, 150.96, 124.62, 117.73, 111.81, 111.26, 102.65, 44.68, 29.76, 25.66, 18.66; HR-ESI-MS *m*/*z*: 300.1232 [M − H]^−^ (calculated for C_17_H_18_NO_4_, 300.1236).

*6-(2-Methyl-acryloylamino)-4-methylumbelliferone* (**4ac**). Pale yellow solid; yield: 53.4%; m.p. 289.1–291.2 °C; ^1^H NMR δ: 11.10 (s, 1H), 8.99 (s, 1H), 8.10 (s, 1H), 6.84 (s, 1H), 6.18 (s, 1H), 5.89 (s, 1H), 5.54 (s, 1H), 2.36 (s, 3H), 1.99 (s, 3H); ^13^C NMR δ: 166.80, 160.71, 153.82, 153.60, 151.73, 140.19, 123.92, 121.26, 119.45, 111.93, 111.35, 102.81, 18.98, 18.67. HR-ESI-MS *m*/*z*: 260.0926 [M + H]^+^ (calculated for C_14_H_14_NO_4_, 260.0923).

*6-Hydrocinnamoylamino-4-methylumbelliferone* (**4ad**). White solid; yield: 59.2%; m.p. 245.5–247.1 °C; ^1^H NMR δ: 11.10 (s, 1H),9.37 (s, 1H), 8.24 (s, 1H), 7.31–7.26 (m, 4H), 7.21–7.17 (m, 1H), 6.84 (s, 1H), 6.16 (s, 1H), 2.91 (t, *J* = 20.8 Hz, 2H), 2.75 (s, *J* = 18.75 Hz, 2H), 2.34 (s, 3H); ^13^C NMR δ: 171.55, 160.80, 153.86, 152.73, 151.07, 141.64, 128.78, 126.41, 124.49, 117.91, 111.68, 111.16, 102.66, 37.93, 31.41, 18.66. HR-ESI-MS *m*/*z*: 324.1235 [M + H]^+^ (calculated for C_19_H_18_NO_4_, 324.1236). 

*6-(4-Methyl-benzoylamino)-4-methylumbelliferone* (**4ae**). White solid; yield: 44.7%; m.p. 286.8–288.1 °C; ^1^H NMR δ: 11.09 (s, 1H), 9.54 (s, 1H), 8.07 (s, 1H), 7.90 (s, 2H), 7.34 (s, 2H), 6.88 (s, 1H), 6.19 (s, 1H), 2.38 (s, 6H); ^13^C NMR δ: 165.72, 160.74, 154.44, 153.86, 152.05, 142.30, 129.54, 128.07, 123.94, 120.81, 111.99, 111.31, 103.01, 21.51, 18.69; HR-ESI-MS *m*/*z*: 308.0923 [M − H]^−^(calculated for C_18_H_14_NO_4_, 308.0923).

*6-(3-Fluoro-benzoylamino)-4-methylumbelliferone* (**4af**). Yellow solid; yield: 49.2%; m.p. 228.8–229.5 °C; ^1^H NMR δ: 11.00 (s, 1H), 9.77 (s, 1H), 7.98 (s, 1H), 7.85 (d, *J* = 7.8 Hz, 1H), 7.80 (d, *J* = 9.9 Hz, 1H), 7.60 (qd, *J*_1_ = 6.0 Hz, *J*_2_ = 2.0 Hz, 1H), 7.46 (td, *J*_1_ = 8.5 Hz, *J*_2_ = 2.0 Hz, 1H), 6.88 (s, 1H), 6.20 (s, 1H), 2.38 (s, 3H); ^13^C NMR δ: 164.66, 162.49, 160.71, 155.04, 153.84, 152.45, 137.09, 131.17, 124.31, 123.39, 121.96, 119.12, 114.99, 112.06, 111.37, 103.12, 18.70; HR-ESI-MS *m*/*z*: 314.0828 [M + H]^+^ (calculated for C_17_H_13_FNO_4_, 314.0829).

*6-(Furyl-2-formylamino)-4-methylumbelliferone* (**4ag**). White solid; yield: 43.4%; m.p. 298.5–299.7 °C; ^1^H NMR δ: 11.23 (s, 1H), 9.27 (s, 1H), 8.21 (s, 1H), 7.95 (s, 1H), 7.31 (s, 1H), 6.88 (s, 1H), 6.72 (s, 1H), 6.20 (s, 1H), 2.37 (s, 3H); ^13^C NMR δ: 160.68, 156.49, 153.78, 153.25, 151.78, 147.75, 146.35, 123.39, 118.94, 115.48, 112.90, 112.05, 111.46, 102.81, 18.63; HR-ESI-MS *m*/*z*: 284.0563 [M − H]^−^ (calculated for C_15_H_10_NO_5_, 284.0559).

*6-(Thienyl-2-formylamino)-4-methylumbelliferone* (**4ah**). White solid; yield: 53.4%; m.p. 253.2–254.8 °C; ^1^H NMR δ: 10.99 (s, 1H), 9.68 (s, 1H), 8.01 (d, *J* = 3.5 Hz, 1H), 7.93 (s, 1H), 7.86 (d, *J* = 4.9 Hz, 1H), 7.22 (t, *J* = 4.5 Hz, 1H), 6.87 (s, 1H), 6.19 (s, 1H), 2.37 (s, 3H); ^13^C NMR δ: 160.66, 160.64, 154.88, 153.77, 152.33, 139.91, 132.27, 129.77, 128.62, 123.20, 121.86, 112.05, 111.33, 103.08, 18.63; HR-ESI-MS *m*/*z*: 300.0328 [M − H]^−^ (calculated for C_15_H_10_NO_4_S, 300.0331).

*8-(n-Butanoylamino)-4-methylumbelliferone* (**4ba**). Pale yellow solid; yield: 17.2%; m.p. 141.1–143.4 °C; ^1^H NMR δ: 10.72(s, 1H), 8.09(s, 1H), 7.36 (d, *J* = 9.0 Hz, 1H), 6.96 (d, *J* = 9.0 Hz, 1H), 6.13 (s, 1H), 2.57 (t, *J* = 7.5 Hz, 2H), 2.42 (s, 3H), 1.86–1.79 (m, 2H), 1.06 (t, *J* = 7.5 Hz, 3H); ^13^C NMR δ: 173.58, 158.83, 152.67, 151.43, 144.56, 121.20, 115.38, 113.00, 111.56, 110.03, 37.86, 18.23, 17.95, 12.56; HR-ESI-MS *m*/*z*: 260.0921 [M − H]^−^ (calculated for C_14_H_14_NO_4_, 260.0923).

*8-(i-Butanoylamino)-4-methylumbelliferone* (**4bb**). White solid; yield: 46.8%; m.p.121.5–122.6 °C; ^1^H NMR δ: 10.83 (s, 1H), 8.18 (s, 1H), 7.36 (d, *J* = 8.9 Hz, 1H), 6.95 (d, *J* = 8.9 Hz, 1H), 6.13 (s, 1H), 2.85–2.81 (m, 1H), 2.42 (s, 3H), 1.34 (d, *J* = 6.9 Hz, 6H); ^13^C NMR δ: 178.65, 159.85, 153.70, 152.46, 145.69, 122.18, 116.37, 114.00, 112.56, 111.02, 36.27, 19.73, 18.96; HR-ESI-MS *m*/*z*: 262.1081 [M + H]^+^ (calculated for C_14_H_16_NO_4_, 262.1079).

*8-(Lauroylamino)-4-methylumbelliferone* (**4bc**). White solid; yield: 25.2%; m.p. 149.2–151.8 °C; ^1^H NMR δ: 10.75 (s, 1H), 8.11 (s, 1H), 7.36 (d, *J* = 8.2 Hz, 1H), 6.96 (d, *J* = 9.4 Hz, 1H), 6.14 (s, 1H), 2.58 (t, *J* = 5.0 Hz, 2H), 2.42 (s, 3H), 1.78 (m, 2H), 1.41–1.26 (m, 16H), 0.88 (t, *J* = 5.0 Hz, 3H); ^13^C NMR δ: 174.87, 159.94, 153.76, 152.47, 145.58, 122.21, 116.44, 114.08, 112.58, 111.04, 37.09, 31.92, 29.60, 29.44, 29.34, 29.28, 29.10, 25.75, 22.70, 18.99, 14.13; HR-ESI-MS *m*/*z*: 396.2146 [M + Na]^+^ (calculated for C_22_H_31_NNaO_4_, 396.2151).

*8-(4-Chloro-butanoylamino)-4-methylumbelliferone* (**4bd**). White solid; yield: 55.7%; m.p. 155.2–156.8 °C; ^1^H NMR δ: 10.41 (s, 1H), 8.30 (s, 1H), 7.36 (d, *J* = 8.8 Hz, 1H), 6.96 (d, *J* = 8.8 Hz, 1H), 6.13 (s, 1H), 3.68 (t, *J* = 6.1 Hz, 2H), 2.82 (t, *J* = 7.1 Hz, 2H), 2.41 (s, 3H), 2.28–2.22 (m, 2H); ^13^C NMR δ: 173.45, 159.90, 153.71, 152.50, 145.73, 122.43, 116.41, 113.90, 112.70, 111.15, 43.92, 33.60, 27.97, 18.96; HR-ESI-MS *m*/*z*: 294.0541, 296.0497 [M − H]^−^ (calculated for C_14_H_13_ClNO_4_, 294.0533, 296.0504).

*8-(Cyclopropyl-formylamino)-4-methylumbelliferone* (**4be**). Yellow solid; yield: 31.2%; m.p. 162.5–163.7 °C; ^1^H NMR δ: 10.82 (s, 1H), 8.40 (s, 1H), 7.34 (d, *J* = 8.9 Hz, 1H), 6.94 (d, *J* = 8.8 Hz, 1H), 6.13 (s, 1H), 2.41 (s, 3H), 1.90–1.86 (m, 1H), 1.22–1.16 (m, 2H), 1.04–1.00 (m, 2H); ^13^C NMR δ: 175.23, 160.08, 153.79, 152.50, 145.46, 121.97, 116.38, 114.34, 112.53, 110.94, 18.95, 15.60, 9.48; HR-ESI-MS *m*/*z*: 260.0921 [M + H]^+^ (calculated for C_14_H_14_NO_4_, 260.0923).

*8-(Cyclohexyl-formylamino)-4-methylumbelliferone* (**4bf**). Yellow solid; yield: 14.1%; m.p. 156.3–158.4 °C; ^1^H NMR δ: 10.87 (s, 1H), 8.12 (s, 1H), 7.3 (d, *J* = 9.0 Hz, 1H), 6.93 (d, *J* = 9.0 Hz, 1H), 6.11 (s, 1H), 2.51 (tt, *J*_1_ = 11.5 Hz, *J*_2_ = 3.5 Hz, 1H), 2.40 (s 3H), 2.01 (d, *J* = 13.5 Hz, 2H), 1.86 (dt, *J*_1_ = 13.5 Hz, *J*_2_ = 3.0 Hz, 2H), 1.58 (qd, *J*_1_ = 12.0 Hz, *J*_2_ = 3.0 Hz, 2H), 1.38–1.24 (m, 4H); ^13^C NMR δ: 177.70, 159.90, 153.73, 152.46, 145.67, 122.13, 116.37, 114.06, 112.54, 111.00, 45.83, 29.74, 25.44, 18.97; HR-ESI-MS *m*/*z*: 302.1384 [M + H]^+^ (calculated for C_17_H_20_NO_4_, 302.1392).

*8-(2-Methyl-acryloylamino)-4-methylumbelliferone* (**4bg**). White solid; yield: 20.8%; m.p. 289.1–291.2 °C; ^1^H NMR δ: 10.75 (s, 1H), 8.44 (s, 1H), 7.35 (d, *J* = 9.0 Hz, 1H), 6.96 (d, *J* = 8.5 Hz, 1H), 6.12 (d, *J* = 7.0 Hz, 2H), 5.66 (d, *J* = 1.0 Hz, 1H), 2.41(s, 3H), 2.17(s, 3H); ^13^C NMR δ: 168.62, 159.57, 153.57, 152.58, 145.90, 138.06, 124.06, 122.41, 116.38, 113.78, 112.62, 111.12, 18.93, 18.59; HR-ESI-MS *m*/*z*: 260.0927 [M + H]^+^ (calculated for C_14_H_14_NO_4_, 260.0923).

*8-(Phenylacetylamino)-4-methylumbelliferone* (**4bh**). White solid; yield: 39.2%; m.p. 219.2–220.9 °C; ^1^H NMR δ: 10.59 (s, 1H), 8.19 (s, 1H), 7.49–7.37 (m, 5H), 7.33 (d, *J* = 8.9 Hz, 1H), 6.93 (d, *J* = 8.8 Hz, 1H), 6.09 (s, 1H), 3.91 (s, 2H), 2.38 (s, 3H); ^13^C NMR δ: 172.56, 159.44, 153.35, 152.29, 145.75, 133.11, 129.52, 129.48, 128.21, 122.34, 116.24, 113.80, 112.57, 111.16, 43.74, 18.89;HR-ESI-MS *m*/*z*: 310.1084 [M + H]^+^ (calculated for C_18_H_16_NO_4_, 310.1079).

*8-(Hydrocinnamoylamino)-4-methylumbelliferone* (**4bi**). White solid; yield: 15.7%; m.p. 237.5–239.0 °C; ^1^H NMR δ: 10.16 (s, 1H),8.17 (s, 1H), 7.35 (d, *J* = 8.8 Hz, 1H), 7.32–7.27 (m, 2H), 7.25–7.17 (m, 3H), 6.95 (d, *J* = 8.8 Hz, 1H), 6.11 (s, 1H), 3.10 (t, *J* = 7.4 Hz, 2H), 2.91 (t, *J* = 7.4 Hz, 2H), 2.40 (s, 3H); ^13^C NMR δ: 172.94, 159.32, 152.98, 151.62, 144.63, 138.71, 127.68, 127.50, 127.30, 127.22, 125.54, 121.25, 115.48, 109.88, 37.36, 30.52, 17.91; HR-ESI-MS *m*/*z*: 322.1077 [M − H]^−^ (calculated for C_19_H_16_NO_4_, 322.1079).

*8-(Cinnamoylamino)-4-methylumbelliferone* (**4bj**). Yellow solid; yield: 41.2%; m.p. 152.3–155.3 °C; ^1^H NMR δ: 11.25 (s, 1H), 8.42 (s, 1H), 7.86 (d, *J* = 15.4 Hz, 1H), 7.61 (s, 2H), 7.49–7.35 (m, 4H), 7.00–6.99 (m, 1H), 6.87 (d, *J* = 15.3 Hz, 1H), 6.16 (s, 1H), 2.44 (s, 3H); ^13^C NMR δ: 166.43, 160.20, 153.98, 152.80, 145.70, 145.43, 134.02, 130.82, 129.09, 128.46, 122.38, 118.27, 116.57, 114.25, 112.57, 110.94, 19.01; HR-ESI-MS *m*/*z*: 344.0901 [M + Na]^+^ (calculated for C_19_H_15_NNaO_4_, 344.0899).

*8-(4-Methyl-benzoylamino)-4-methylumbelliferone* (**4bk**). Yellow solid; yield: 16.8%; m.p. 183.8–185.6 °C; ^1^H NMR δ: 10.93 (s, 1H), 8.78 (s, 1H), 7.89 (d, *J* = 8.2 Hz, 2H), 7.37 (d, *J* = 8.9 Hz, 1H), 7.32 (d, *J* = 8.2 Hz, 2H), 6.98 (d, *J* = 8.8 Hz, 1H), 6.12 (s, 1H), 2.44 (s, 3H), 2.41 (s, 3H); ^13^C NMR δ: 167.85, 159.72, 153.64, 152.68, 146.07, 144.08, 129.84, 129.35, 127.78, 122.35, 116.44, 114.12, 112.69, 111.13, 21.67, 18.97; HR-ESI-MS *m*/*z*: 332.0899 [M + Na]^+^ (calculated for C_18_H_15_NNaO_4_, 332.0899).

*8-(4-Methoxyl-benzoylamino)-4-methylumbelliferone* (**4bl**). White solid; yield: 43.4%; m.p. 151.2–154.2 °C; ^1^H NMR δ: 11.08 (s, 1H), 8.69 (s, 1H), 7.98 (d, *J* = 8.9 Hz, 2H), 7.39 (d, *J* = 8.9 Hz, 1H), 7.03 (d, *J* = 9.0, 2H), 7.01 (d, *J* = 8.5 Hz, 1H), 6.15 (s, 1H), 3.90 (s, 3H), 2.44 (s, 3H); ^13^C NMR δ: 167.34, 163.61, 159.74, 153.70, 152.57, 145.95, 129.81, 124.26, 122.21, 116.46, 114.45, 114.21, 112.66, 111.10, 55.63, 29.72; HR-ESI-MS *m*/*z*: 326.1036 [M + H]^+^ (calculated for C_18_H_16_NO_5_, 326.1028).

*8-(3-Fluoro-benzoylamino)-4-methylumbelliferone* (**4bm**). White solid; yield: 26.8%; m.p. 261.7–263.2 °C; ^1^H NMR δ: 10.52 (s, 1H), 8.79 (s, 1H), 7.77 (dq, *J*_1_ = 7.8 Hz, *J*_2_ = 0.5 Hz, 1H), 7.72 (dt, *J*_1_ = 8.8 Hz, *J*_2_ = 2.5 Hz, 1H), 7.56–7.52 (m, 1H), 7.42 (d, *J* = 8.9 Hz, 1H), 7.34 (td, *J*_1_ = 8.6, *J*_2_ = 3.3 Hz, 1H), 7.02 (d, *J* = 8.9 Hz, 1H), 6.16 (s, 1H), 2.44 (s, 3H); ^13^C NMR δ: 166.61, 162.94 (*J* = 249.48 Hz), 159.61, 153.66, 152.65, 146.15, 134.47 (*J* = 7.56 Hz), 130.97 (*J* = 8.82 Hz), 123.00 (*J* = 2.52 Hz), 122.77, 120.31 (*J* = 21.42 Hz), 116.57, 115.42 (*J* = 23.94 Hz), 113.78, 112.84, 111.28, 18.99; HR-ESI-MS *m*/*z*: 312.0676 [M − H]^−^ (calculated for C_17_H_11_FNO_4_, 312.0672).

*8-(3-Bromo-benzoylamino)-4-methylumbelliferone* (**4bn**). White solid; yield: 40.7%; m.p. 237.2–239.3 °C; ^1^H NMR δ: 10.44 (s, 1H), 8.78 (s, 1H), 8.17 (s, 1H), 7.90 (d, *J* = 7.5 Hz, 1H), 7.76 (d, *J* = 7.8 Hz, 1H), 7.49–7.37 (m, 2H), 7.03 (d, *J* = 8.8 Hz, 1H), 6.16 (s, 1H), 2.44 (s, 3H); ^13^C NMR δ: 166.51, 159.62, 153.66, 152.65, 146.16, 136.14, 134.20, 131.27, 130.63, 125.83, 123.45, 122.81, 116.60, 113.78, 112.86, 111.30, 19.00; HR-ESI-MS *m*/*z*: 374.0025, 376.0016 [M + H]^+^ (calculated for C_17_H_13_BrNO_4_, 374.0028, 376.0007).

*8-(Furyl-2-formylamino)-4-methylumbelliferone* (**4bo**). White solid; yield: 23.4%; m.p. 280.1–281.5 °C; ^1^H NMR δ: 10.77 (s, 1H), 8.96 (s, 1H), 7.67 (s, 1H), 7.40 (d, *J* = 9.0 Hz, 1H), 7.37 (d, *J* = 3.5 Hz, 1H), 7.00 (d, *J* = 9.0 Hz, 1H), 6.63 (s, 1H), 6.16 (s, 1H), 2.43 (s, 3H); ^13^C NMR δ: 159.61, 157.82, 153.45, 152.34, 146.17, 145.99, 145.94, 122.43, 117.76, 116.40, 113.57, 113.05, 112.77, 111.30, 18.92;HR-ESI-MS *m*/*z*: 284.0564 [M − H]^−^ (calculated for C_15_H_10_NO_5_, 284.0559).

*8-(Thienyl-2-formylamino)-4-methylumbelliferone* (**4bp**). White solid; yield: 29.2%; m.p. 244.6–246.2 °C; ^1^H NMR δ: 10.67 (s, 1H), 8.66 (s, 1H), 7.86 (d, *J* = 3.8 Hz, 1H), 7.68 (d, *J* = 4.9 Hz, 1H), 7.40 (d, *J* = 8.9 Hz, 1H), 7.20 (t, *J* = 4.5 Hz, 1H), 7.01 (d, *J* = 8.9 Hz, 1H), 6.15 (s, 1H), 2.43 (s, 3H); ^13^C NMR δ: 162.21, 159.60, 153.63, 152.36, 145.84, 136.42, 133.09, 130.56, 128.44, 122.42, 116.47, 113.81, 112.75, 111.20, 18.97; HR-ESI-MS *m*/*z*: 302.0491 [M + H]^+^ (calculated for C_15_H_12_NO_4_S, 302.0487).

*8-(Naphthyl-1-formylamino)-4-methylumbelliferone* (**4bq**). White solid; yield: 41.2%; m.p. 208.5–210.9 °C; ^1^H NMR δ: 10.69 (s, 1H), 8.63 (s, 1H), 8.44 (d, *J* = 8.4 Hz, 1H), 8.07 (d, *J* = 8.3 Hz, 1H), 7.94 (t, *J* = 7.3 Hz, 2H), 7.66 (t, *J* = 7.0 Hz, 1H), 7.59 (q, *J* = 7.5 Hz, 2H), 7.45 (d, *J* = 8.9 Hz, 1H), 7.08 (d, *J* = 8.9 Hz, 1H), 6.15 (s, 1H), 2.45 (s, 3H); ^13^C NMR δ: 170.31, 159.70, 153.60, 152.68, 146.12, 133.87, 132.64, 131.46, 130.06, 128.72, 128.02, 126.97, 126.61, 124.92, 124.80, 122.71, 116.61, 114.32, 112.89, 111.30, 19.00; HR-ESI-MS *m*/*z*: 346.1079 [M + H]^+^ (calculated for C_21_H_16_NO_4_, 346.1079).

### 3.2. Procedures for ActivityEvaluation

#### 3.2.1. Acaricidal Activity

The acaricidal activities of the title compounds against the phytophagous mite *T. cinnabarinus* were evaluated by the microimmersion bioassay method [45]. Each title compound was dissolved by acetone (for **4ba**–**4bq**) or DMSO/H_2_O (1:1, for **4aa**–**4ah**) to give a 10 g/L stock solution. Then 0.1 and 0.5 mL of the stock solution were diluted separately to 5 mL by an appropriate amount of acetone (DMSO/H_2_O) and 0.1% Tween 80 in order to achieve the 0.2 g/L and 1 g/L, respectively, of test compound in acetone (DMSO/H_2_O)-0.1% Tween 80 (3:7). Ten test mites were sucked into the terminal pipette tip by a sucking trap, then 150 μL of the compound solution was sucked in to immerse the mites for 15 seconds. After the exposure, the mites were carefully removed onto a piece of filter paper in order to air dry the body surface. At the same time, the petiole of a fresh peanut leaf was wrapped around a ball of absorbent cotton, which was then wetted and sealed with a piece of silver paper. The hydrated leaf was dipped into the same compound solution for 5 s and taken out to air dry, as with the mites. Then the treated mites and peanut leaf were sealed in one well of a six pore plate with a piece of rice paper. Finally the mites were reared in an illumination incubator set at 28 ± 1 °C and 50–55% relative humidity in alternating 12 h of light and 12 h of dark for 72 h. Acetone (DMSO/H_2_O)-0.1% Tween 80 (3:7) was used as a blank control, and Bifenazate was used as a positive control. Each treatment was conducted in four replicates. The dead mites were recorded every 24 h under a stereoscope and the corrected mortalities of *T. cinnabarinus* treated by the compounds were calculated.

#### 3.2.2. Herbicidal Activity

The herbicidal activities of the synthesized compounds against the taproot and caulis development of the dicotyledonous weed *C. album* and the monocotyledonous weed *D. sanguinalis* were determined in vitro [38]. A suspension of 5 g agar powder in 1 L distilled water was heated to melt, and then cooled to 40–50 °C. Each title compound was dissolved by acetone to give a stock solution of 10 g/L, and 0.1 or 0.5 mL of the stock solution was added to 50 mL of the melting agar at 45 °C to achieve the required concentrations. Then, 5 mL of the agar-containing compound was added to a beaker (10 mL) and cooled, and uniform germinating seeds were placed on the surface of the agar mass. The beaker was sealed by a piece of plastic wrap with several small holes on it, and then the cultivations were carried out in an illumination incubator set at 28 ± 1 °C and 50–55% relative humidity in alternating 12 h of light and 12 h of dark for 3 days. Acetone was used as a blank control, and Acetochlor was used as a positive control. Each treatment was conducted in three replicates. After 3 days of cultivation, the taproot and caulis lengths were measured, and the growth inhibitory rate related to the untreated control was determined.

#### 3.2.3. Antifungal Activity

The antifungal activities of the title compounds against *B. cinerea*, *C. gloeosporioides*, *A. brassicae* and *G. gramini* were tested in vitro using the mycelium growth rate test [46]. The test compound was dissolved in acetone to form a series of proper concentration solutions. Then 1 mL of the solution was added to 100 mL melting potato dextrose agar (PDA) at 45 °C, and the mixture was shaken up to obtain the required concentration of the poisoned medium. 5 mL of the poisoned medium was poured into a 6 cm petri dish and cooled to room temperature to get a solid plate. After that, a 4 mm activated mycelium disk was inoculated on the PDA plate and incubated in the dark at 28 °C for 48 h. The mycelium elongation radius (mm) of the fungus settlements was measured and the growth inhibition rate related to the untreated control was calculated. Acetone was used as a blank control, and Carbendazim was used as a positive control. Each treatment was repeated for 3 times.

## 4. Conclusions

Twenty-five 4-methylumbelliferone amide derivatives were synthesized and their acaricidal, herbicidal and antifungal activities were evaluated. Compounds **4bi** [8-(hydrocinnamoylamino)], **4ac** [6-(2-methyl-acryloylamino)] and **4bd** [8-(4-chloro-butanoylamino)] were strongly acaricidal against *T. cinnabarinus*, with 72 h corrected mortalities of greater than 80% at 1000 mg/L. Compounds **4bh** [8-(phenylacetylamino)] and **4bf** [8-(cyclohexyl-formylamino)] exhibited the strongest inhibition against the taproot development of *D. sanguinalis* and *C. glaucum*, and were more potent than the commercial herbicide Acetochlor to *D. sanguinalis*. Moreover, compounds **4bk** [8-(4-methyl-benzoylamino)], **4bh** [8-(phenylacetylamino)] and **4bp** [8-(thienyl-2-formylamino)] showed the highest antifungal activities against the mycelium growth of *V. mali*, and were more effective than the commercial fungicide Carbendazim.

## Figures and Tables

**Table:** 

Compd.	R	Compd.	R	Compd.	R
**4aa**	-CH_2_CH_2_CH_3_	**4ba**	-CH_2_CH_2_CH_3_	**4bi**	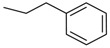
**4ab**	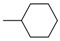	**4bb**	-CH(CH_3_)CH_3_	**4bj**	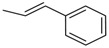
**4ac**	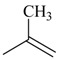	**4bc**	-CH_2_(CH_2_)_9_CH_3_	**4bk**	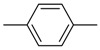
**4ad**	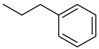	**4bd**	-CH_2_CH_2_CH_2_Cl	**4bl**	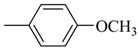
**4ae**	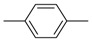	**4be**	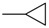	**4bm**	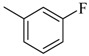
**4af**	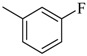	**4bf**	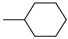	**4bn**	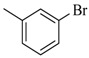
**4ag**	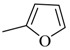	**4bg**	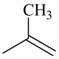	**4bo**	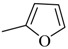
**4ah**	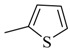	**4bh**	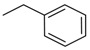	**4bp**	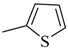
				**4bq**	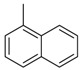

**Table 1:** Acaricidal activities of title compounds against *T. cinnabarinus* (%, 72 h corrected mortality).

Compd.	200 mg/L	1000 mg/L	Compd.	200 mg/L	1000 mg/L
**4aa**	4.2 ± 5.2	12.9 ± 5.0	**4bf**	18.4 ± 3.0	29.2 ± 6.3
**4ab**	4.5 ± 3.2	27.1 ± 6.5	**4bg**	40.3 ± 3.7	71.7 ± 5.4
**4ac**	51.9 ± 9.9	88.3 ± 4.9	**4bh**	54.0 ± 13.1	65.1 ± 6.6
**4ad**	9.1 ± 5.0	19.4 ± 2.7	**4bi**	87.8 ± 8.1	98.6 ± 6.1
**4ae**	23.0 ± 4.9	42.1 ± 10.9	**4bj**	26.6 ± 5.8	38.0 ± 8.0
**4af**	33.9 ± 2.9	56.9 ± 4.7	**4bk**	57.4 ± 10.9	79.8 ± 8.3
**4ag**	32.8 ± 5.6	52.6 ± 7.3	**4bl**	23.0 ± 6.3	31.7 ± 9.7
**4ah**	30.0 ± 3.5	53.1 ± 7.5	**4bm**	7.1 ± 8.6	55.0 ± 10.7
**4ba**	20.4 ± 9.6	30.0 ± 3.4	**4bn**	47.4 ± 10.3	58.8 ± 7.4
**4bb**	11.8 ± 7.1	29.7 ± 10.0	**4bo**	53.8 ± 8.0	63.4 ± 4.6
**4bc**	61.5 ± 2.4	69.2 ± 2.4	**4bp**	48.7 ± 9.5	62.6 ± 3.0
**4bd**	75.1 ± 7.6	83.5 ± 5.6	**4bq**	41.9 ± 3.0	72.6 ± 4.2
**4be**	65.1 ± 3.2	73.8 ± 2.3	Bifenazate	93.5 ± 5.7	100.0 ± 0.0

**Table 2:** Herbicidal activities of target compounds (%, 100 mg/L).

Compd.	*C. glaucum*		Compd.	*D. sanguinalis*		Compd.	*D. sanguinalis*	
	Taproot	Caulis		Taproot	Caulis		Taproot	Caulis
**4af**	31.2 ± 9.7	-	**4aa**	41.6 ± 2.6	67.4 ± 4.4	**4bj**	10.6 ± 5.7	61.5 ± 12.4
**4ah**	-	40.0 ± 5.3	**4ab**	17.4 ± 7.4	33.6 ± 9.1	**4bk**	41.9 ± 8.5	54.8 ± 8.7
**4bb**	37.1 ± 9.6	16.8 ± 6.5	**4ad**	25.5 ± 4.4	53.1 ± 8.9	**4bl**	27.8 ± 5.2	48.5 ± 7.9
**4bc**	40.1 ± 8.6	15.3 ± 6.5	**4ag**	30.6 ± 3.6	-	**4bm**	44.8 ± 7.7	12.2 ± 13.0
**4be**	35.2 ± 3.4	-	**4ba**	57.1 ± 4.3	70.1 ± 5.9	**4bo**	64.6 ± 4.4	17.7 ± 5.1
**4bf**	61.1 ± 9.7	50.6 ± 4.8	**4bb**	66.7 ± 5.1	49.0 ± 12.8	**4bp**	15.5 ± 3.3	42.5 ± 12.3
**4bh**	76.3 ± 3.3	39.3 ± 1.9	**4bc**	-	45.4 ± 11.7	**4bq**	54.8 ± 4.3	60.9 ± 10.9
**4bk**	41.8 ± 6.0	-	**4bd**	3.4 ± 12.1	62.6 ± 10.4	Acetochlor	84.2 ± 7.0	78.1 ± 8.4
**4bm**	51.1 ± 7.9	-	**4be**	18.9 ± 6.8	40.5 ± 5.6			
**4bn**	31.1 ± 2.2	10.9 ± 8.1	**4bf**	86.4 ± 10.6	71.8 ± 8.1			
**4bo**	30.1 ± 2.6	63.0 ± 5.8	**4bg**	44.4 ± 11.7	-			
**4bq**	41.8 ± 6.0	9.4 ± 10.5	**4bh**	96.2 ± 7.0	52.0 ± 6.4			
Acetochlor	88.2 ± 2.1	78.5 ± 7.0	**4bi**	48.0 ± 6.7	47.7 ± 10.2			

**Table 3:** Antifungal activities of target compounds (%, 100 mg/L).

Compd.	F1	Compd.	F2	Compd.	F3	Compd.	F4	Compd.	F4
**4bf**	45.8± 8.6	**4ab**	48.2± 7.4	**4ac**	31.8± 7.9	**4ab**	32.6± 1.1	**4bj**	39.8± 2.1
**4bh**	32.1±5.5	**4ag**	45.9± 9.0	**4bh**	38.4± 3.3	**4ae**	32.6± 8.9	**4bk**	88.7± 1.9
**4bk**	56.6± 5.0	**4bd**	54.6± 7.8	**4bj**	36.1± 4.2	**4ba**	47.3± 1.6	**4bl**	48.1± 2.2
**4bl**	39.3± 6.1	**4be**	69.4± 6.2	**4bk**	46.6± 1.9	**4bd**	36.5± 1.6	**4bm**	51.0± 2.5
**4bq**	60.8± 3.6	**4bf**	32.9± 9.8	**4bn**	48.9± 4.1	**4bf**	46.5± 4.0	**4bn**	72.7± 4.1
Carb.	88.9± 4.7	**4bi**	57.8± 3.8	**4bp**	47.9± 4.2	**4bg**	66.1± 5.2	**4bo**	34.8± 8.6
		**4bl**	34.5± 9.9	**4bq**	71.2± 4.8	**4bh**	84.9± 1.6	**4bp**	81.8± 4.2
		**4bn**	37.7± 6.8	Carb.	70.9± 8.4	**4bi**	58.4±2.7	**4bq**	61.1± 4.8
		Carb.	54.1± 7.1					Carb.	76.1± 3.7

F1: *C. glecosporioides*; F2: *B. cinerea*; F3: *F. oxysporum*; F4: *V. mali*; Carb.: Carbendazim.

## Supplementary Materials

The following are available online.
