# Supplementary material for: Novel 4-Methylumbelliferone Amide Derivatives: Synthesis, Characterization and Pesticidal Activities
Source: Molecules. 2018 Jan 8;23(1):122. doi: 10.3390/molecules23010122 (PMC6017845; doi:10.3390/molecules23010122)
Supplement: Supplementary file 1 [file molecules-23-00122-s001.pdf]

Supplementary Materials

The ^{1}H-NMR Spectra of Compounds **4aa-4bq**.

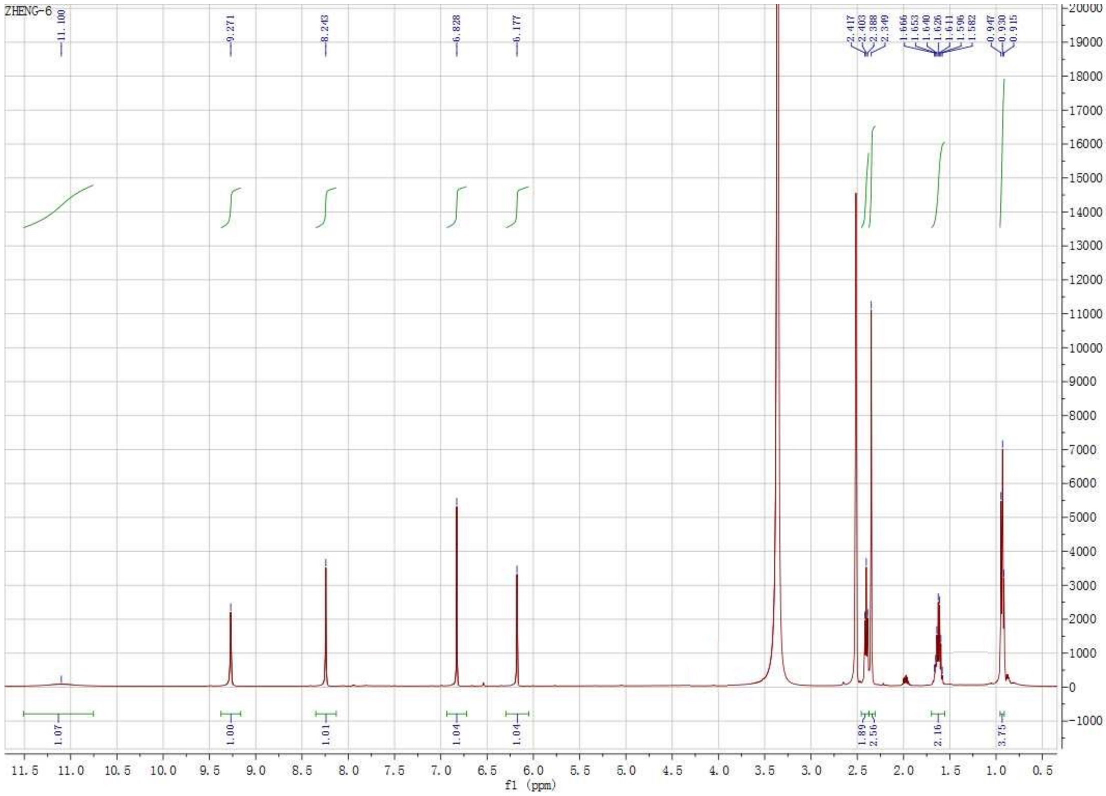

Figure S1. ^{1}H NMR of **4aa**

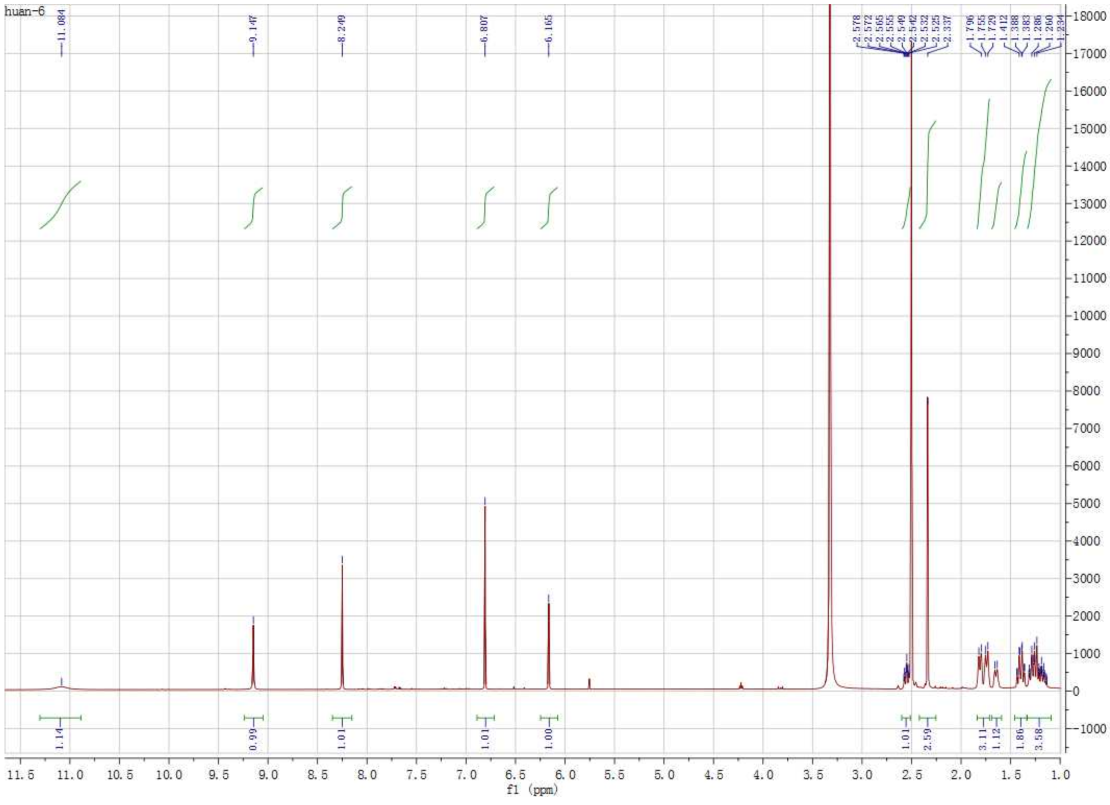

Figure S2. ^{1}H NMR of **4ab**

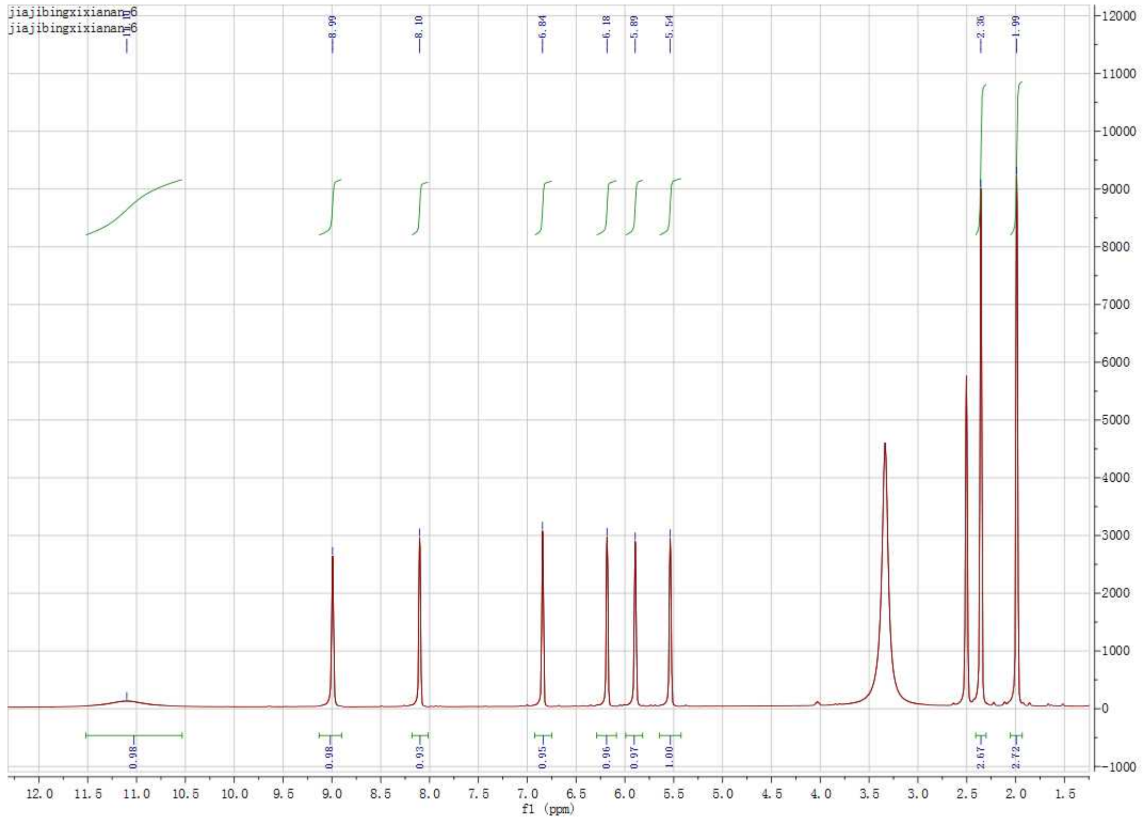

Figure S3. ^{1}H NMR of **4ac**

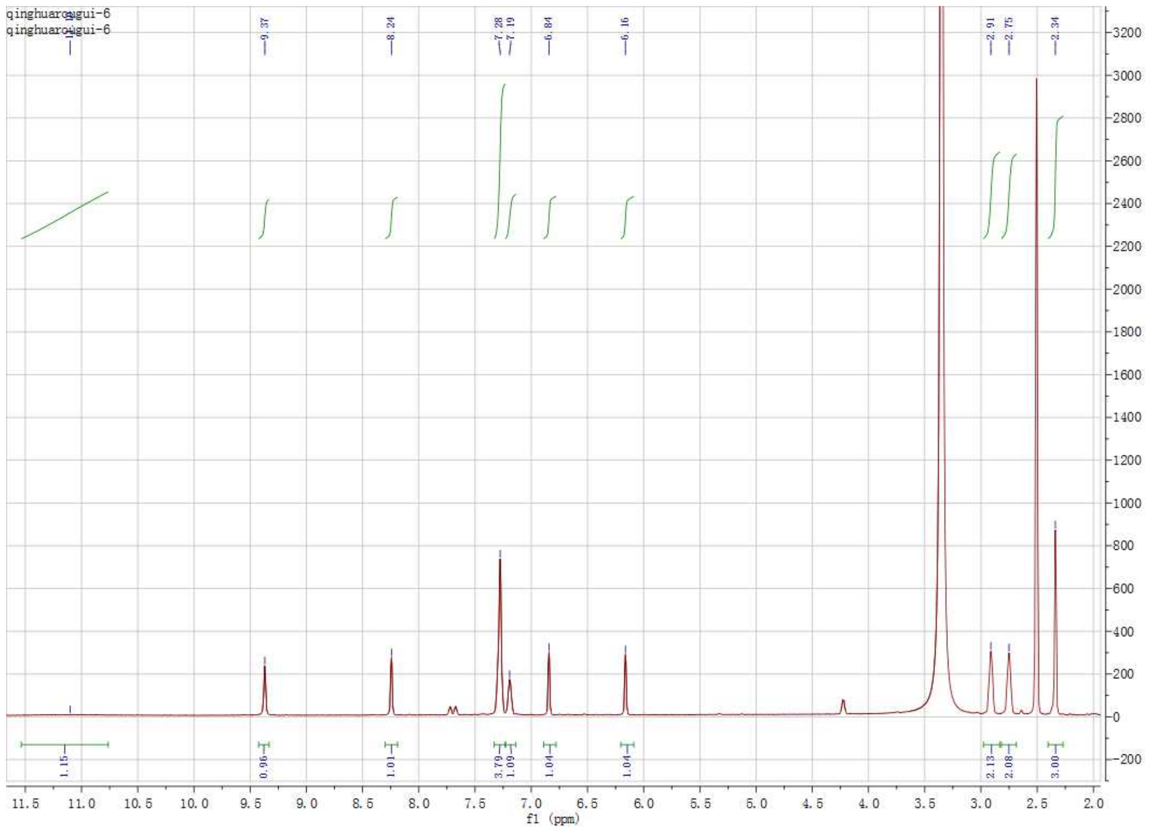

Figure S4. ^{1}H NMR of **4ad**

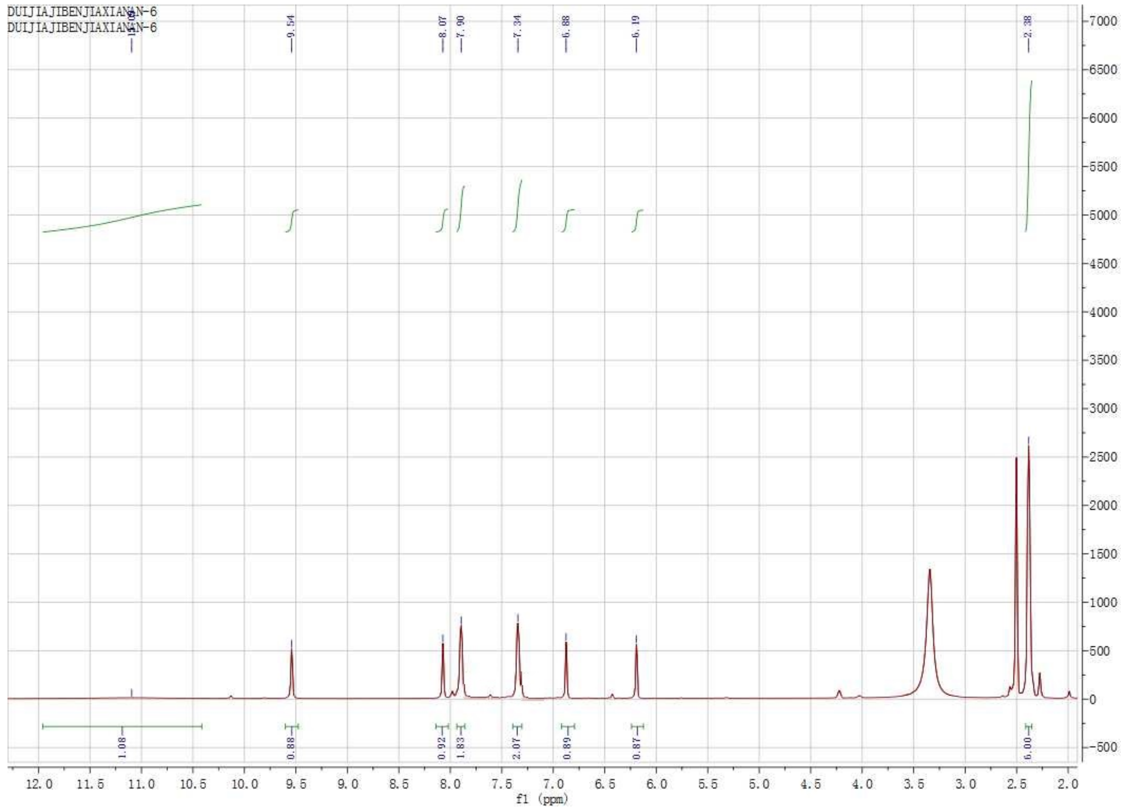

Figure S5. ^{1}H NMR of **4ae**

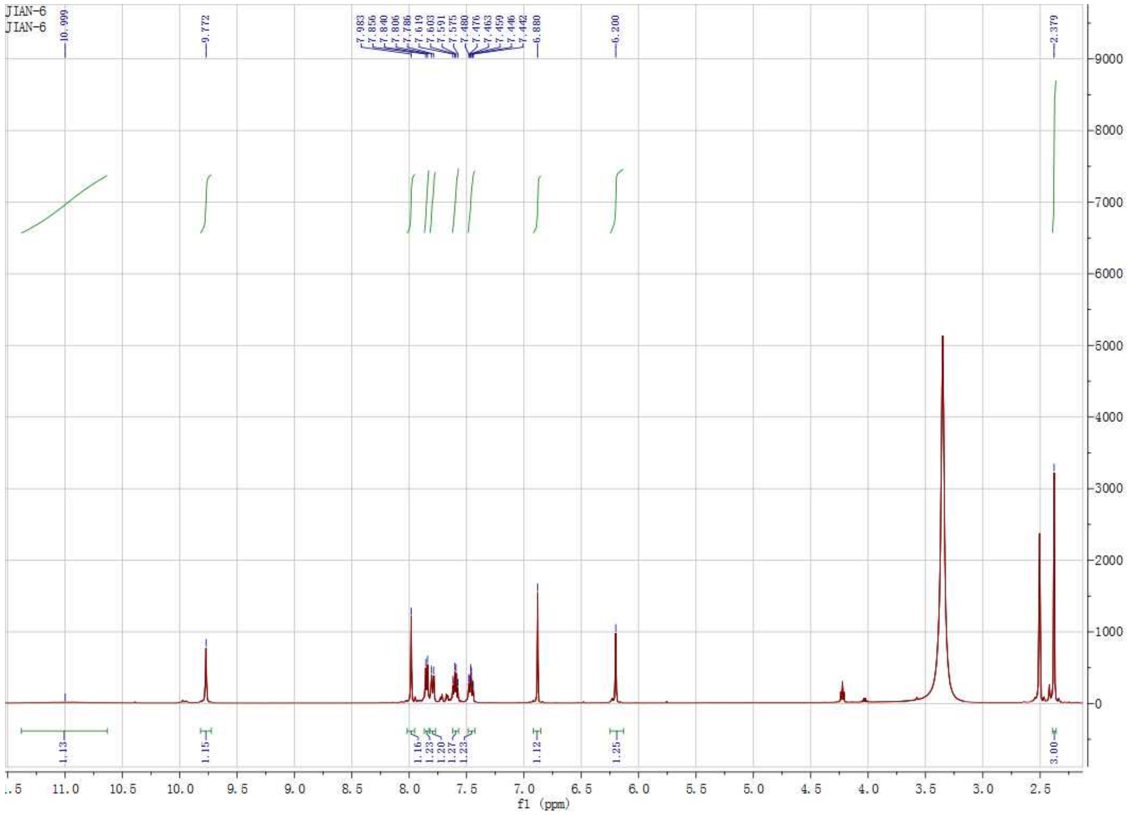

Figure S6. ^{1}H NMR of **4af**

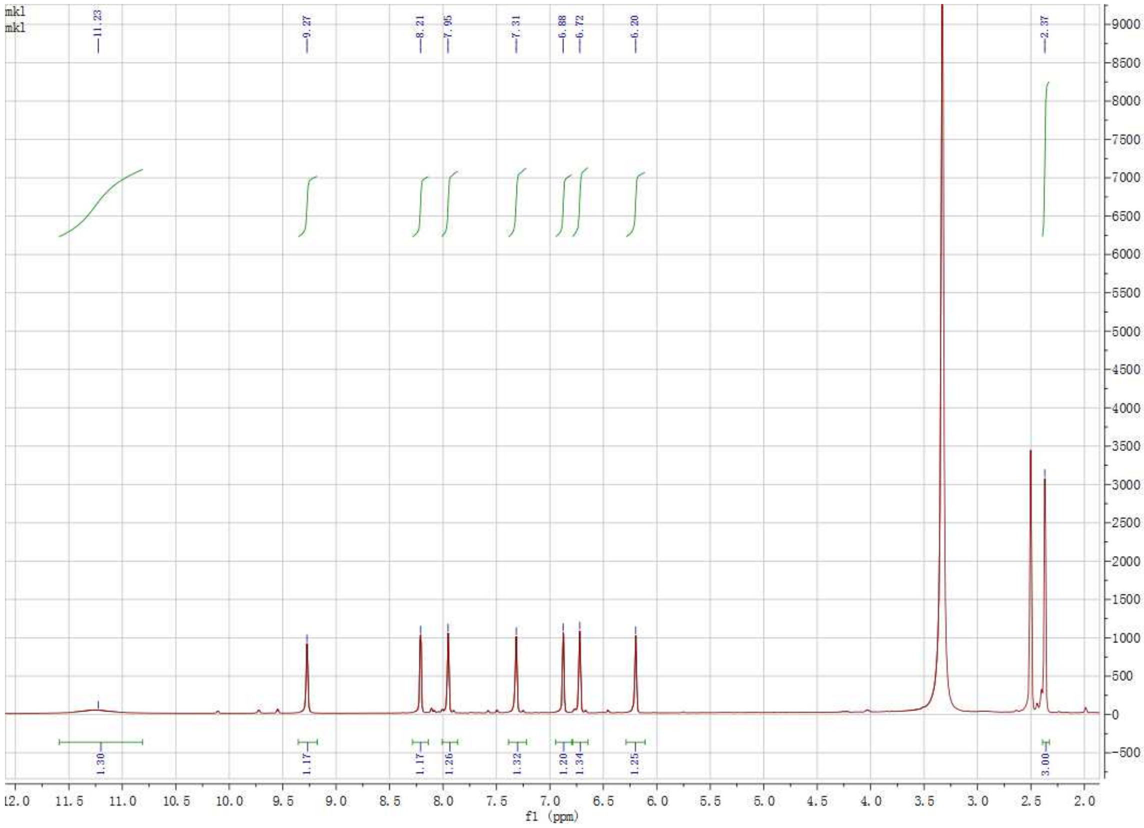

Figure S7. ^{1}H NMR of **4ag**

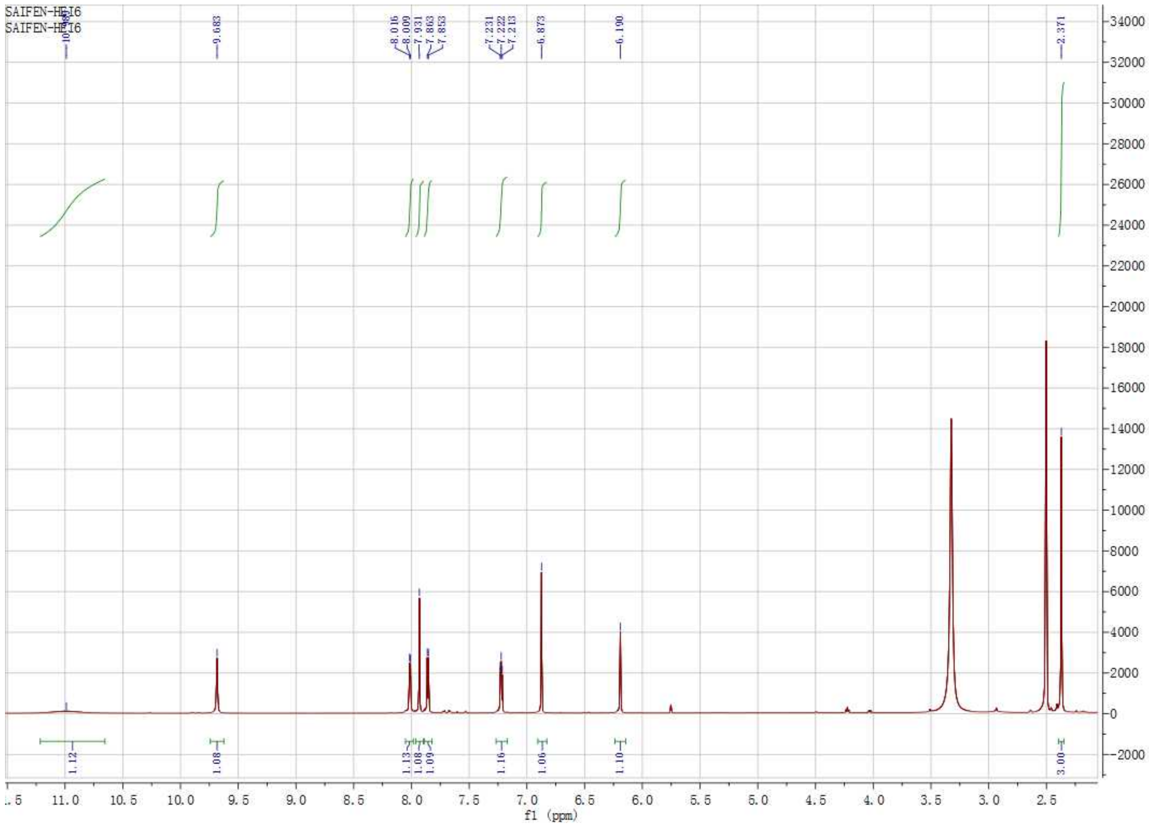

Figure S8. ^{1}H NMR of **4ah**

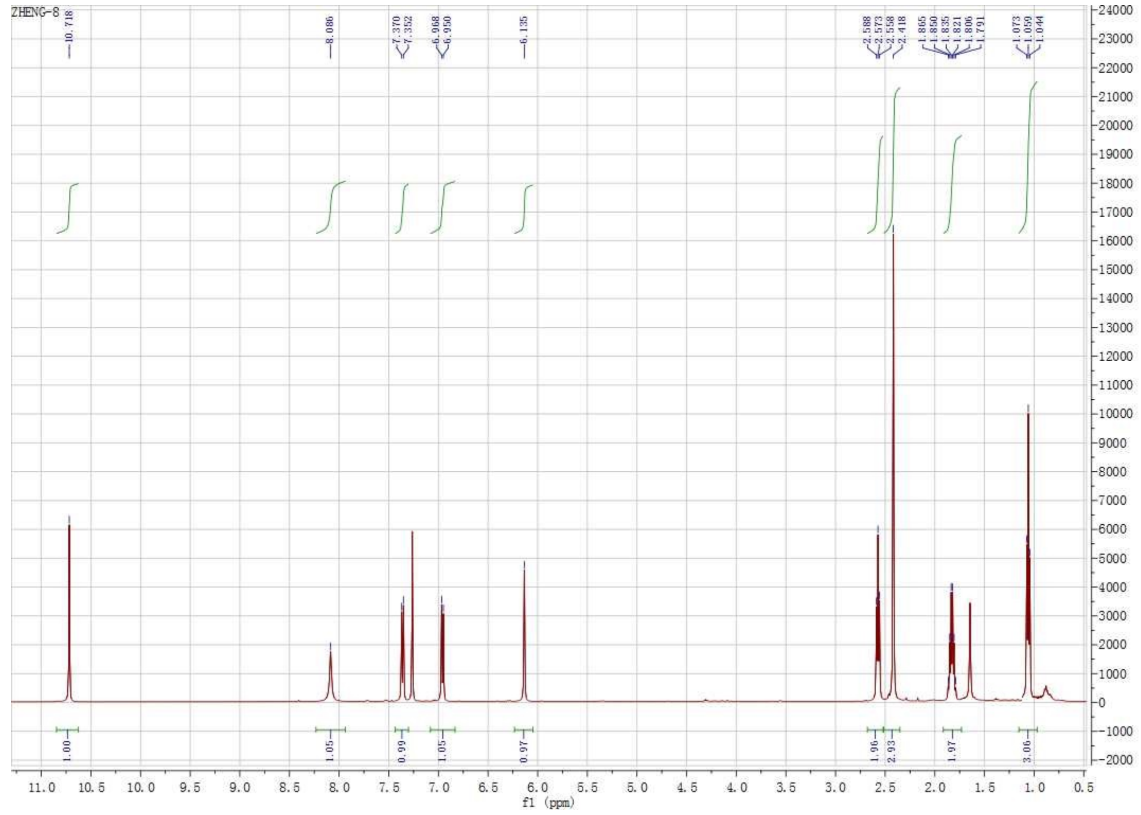

Figure S9. ^{1}H NMR of **4ba**

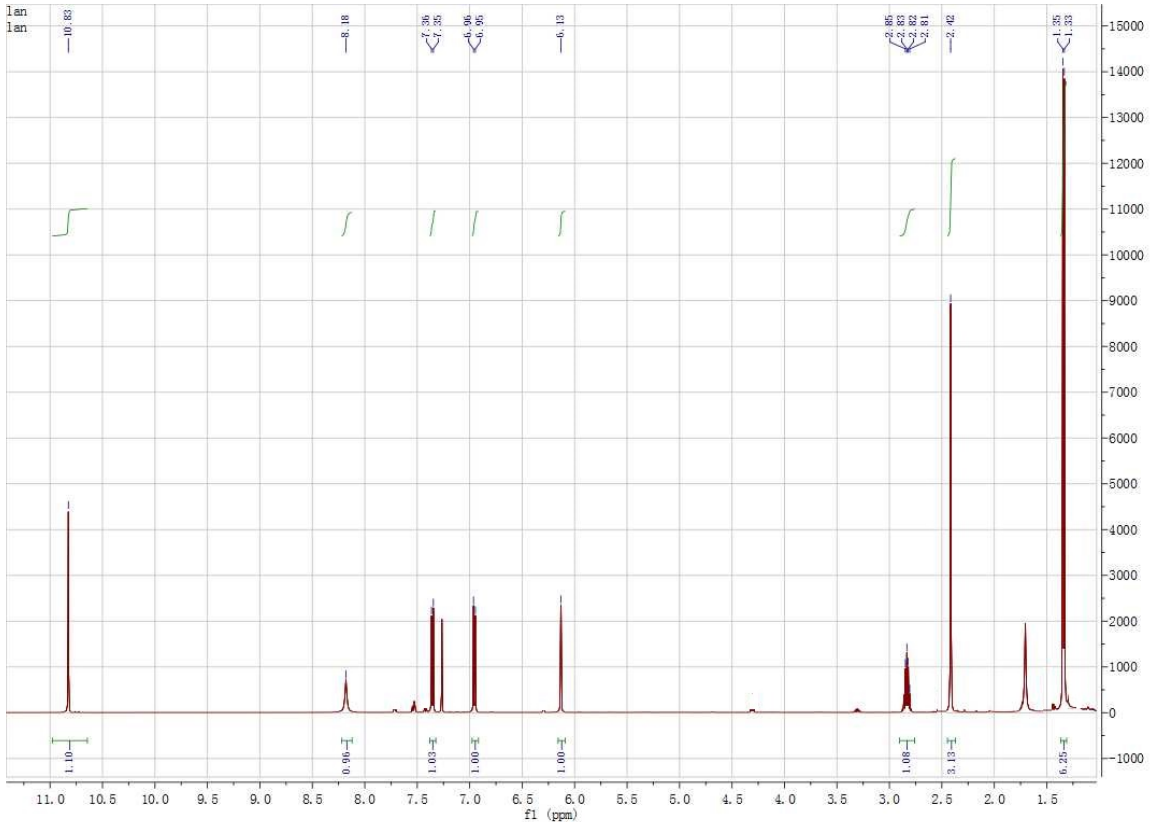

Figure S10. ^{1}H NMR of **4bb**

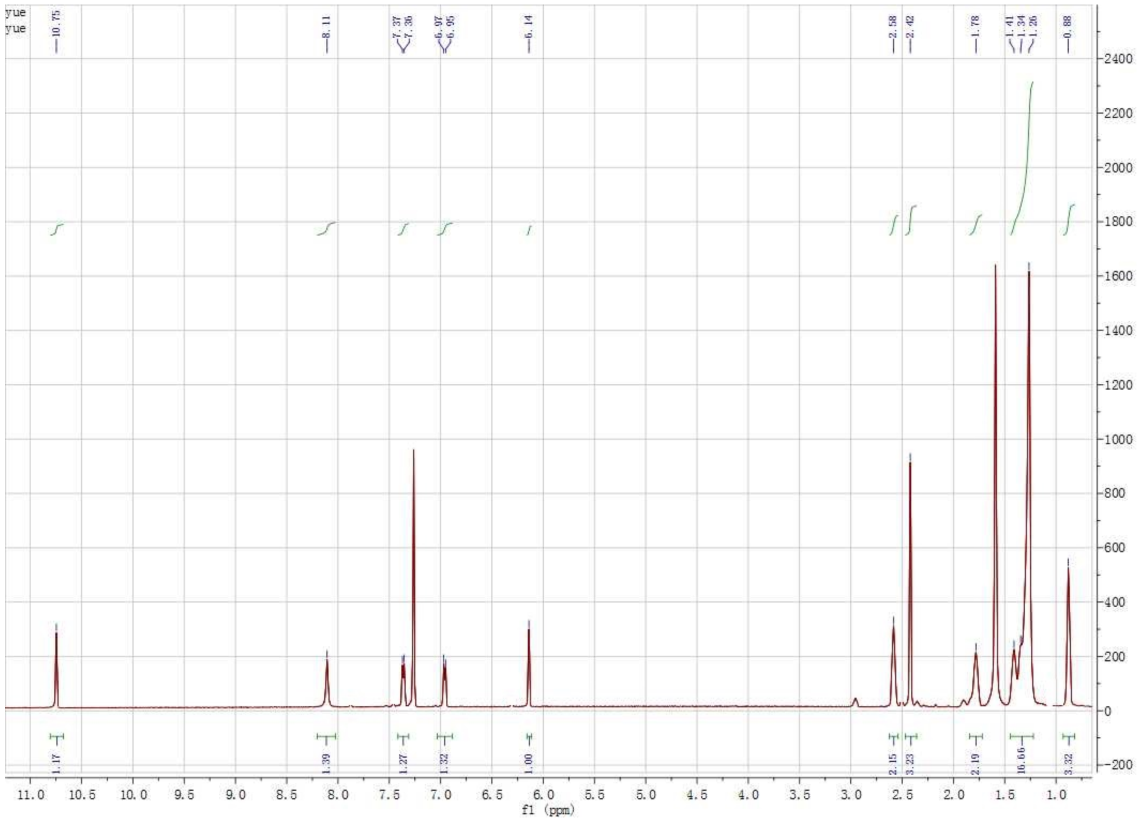

Figure S11. ^{1}H NMR of **4bc**

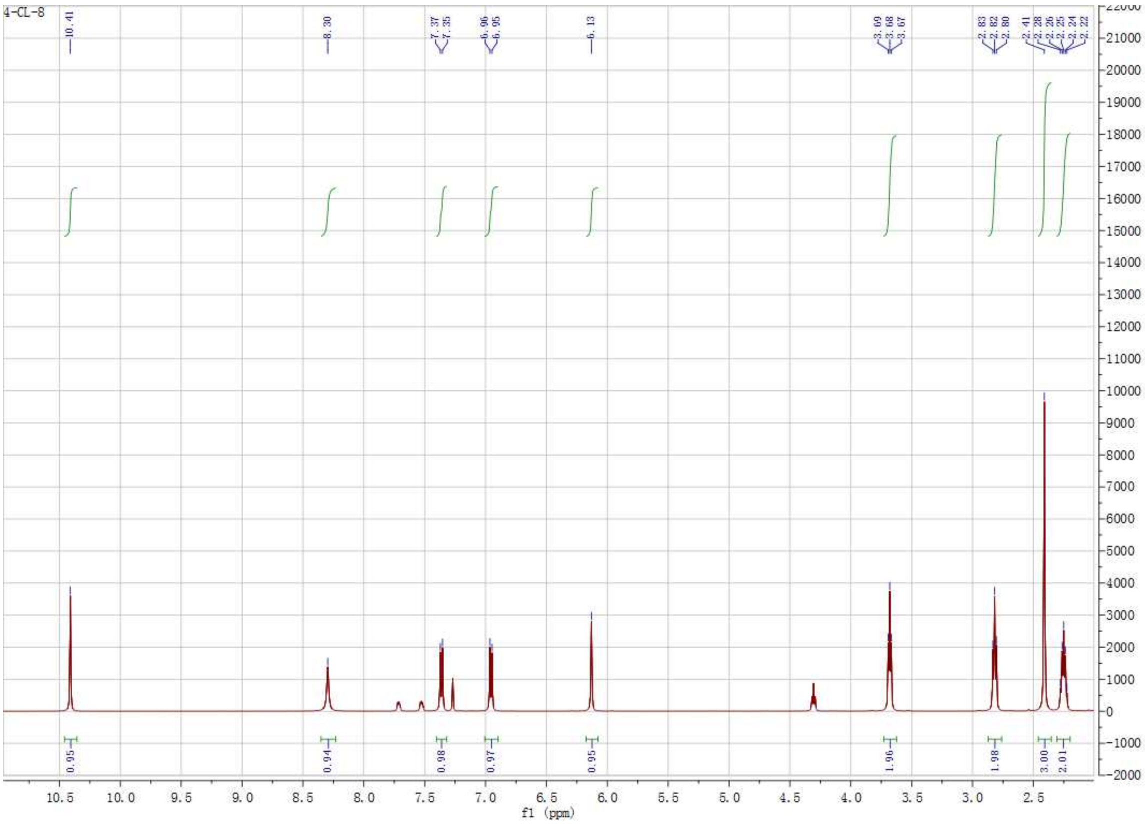

Figure S12. ^{1}H NMR of **4bd**

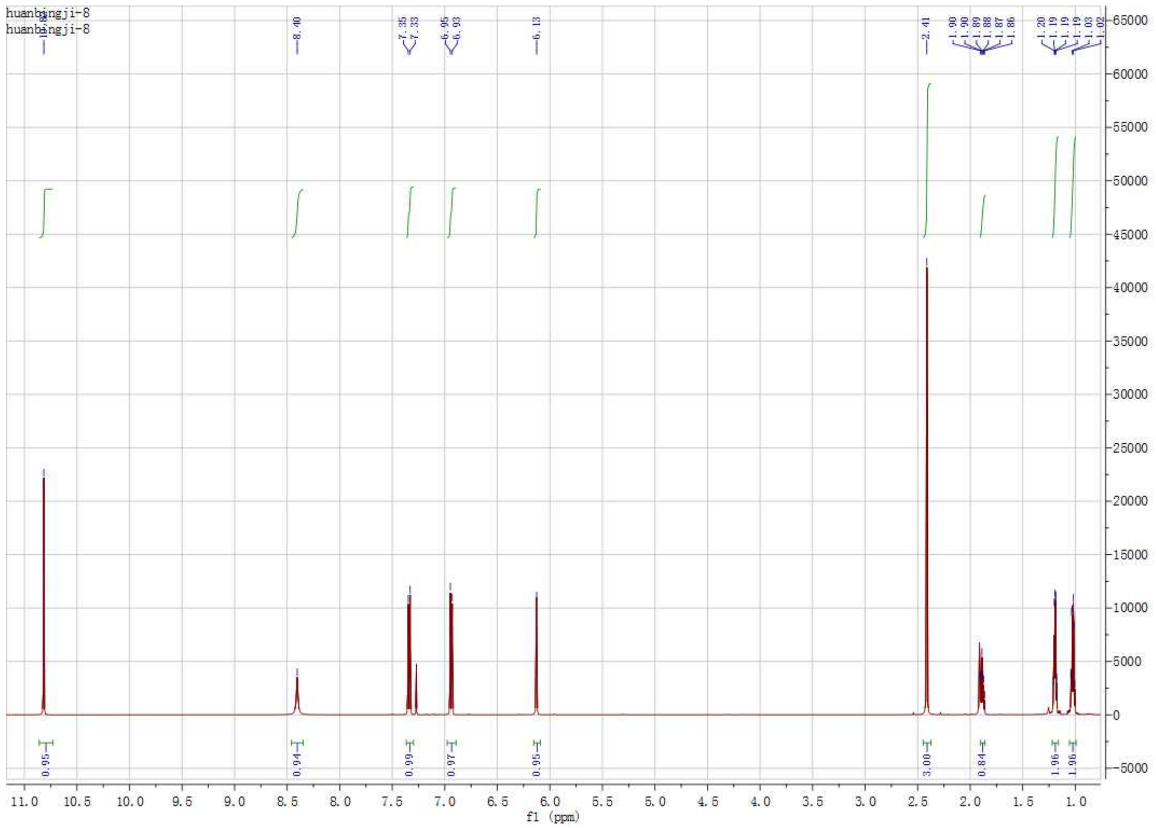

Figure S13. ^{1}H NMR of **4be**

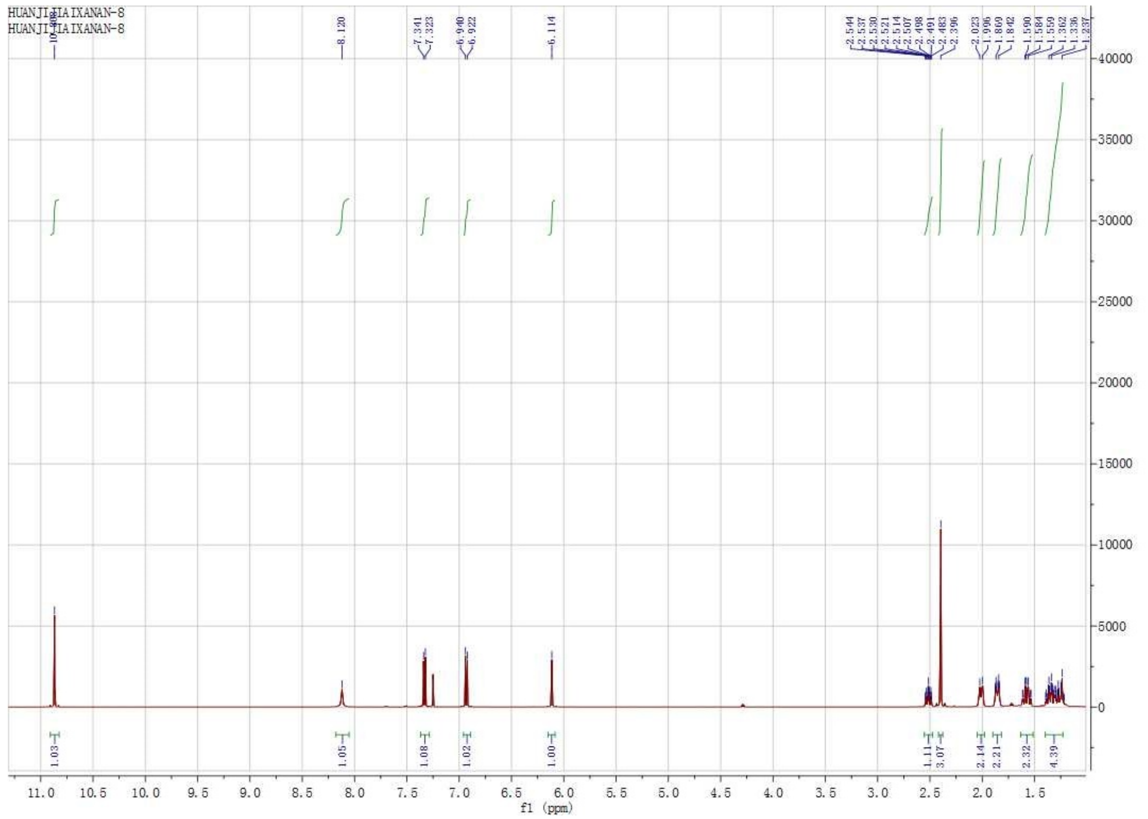

Figure S14. ^{1}H NMR of **4bf**

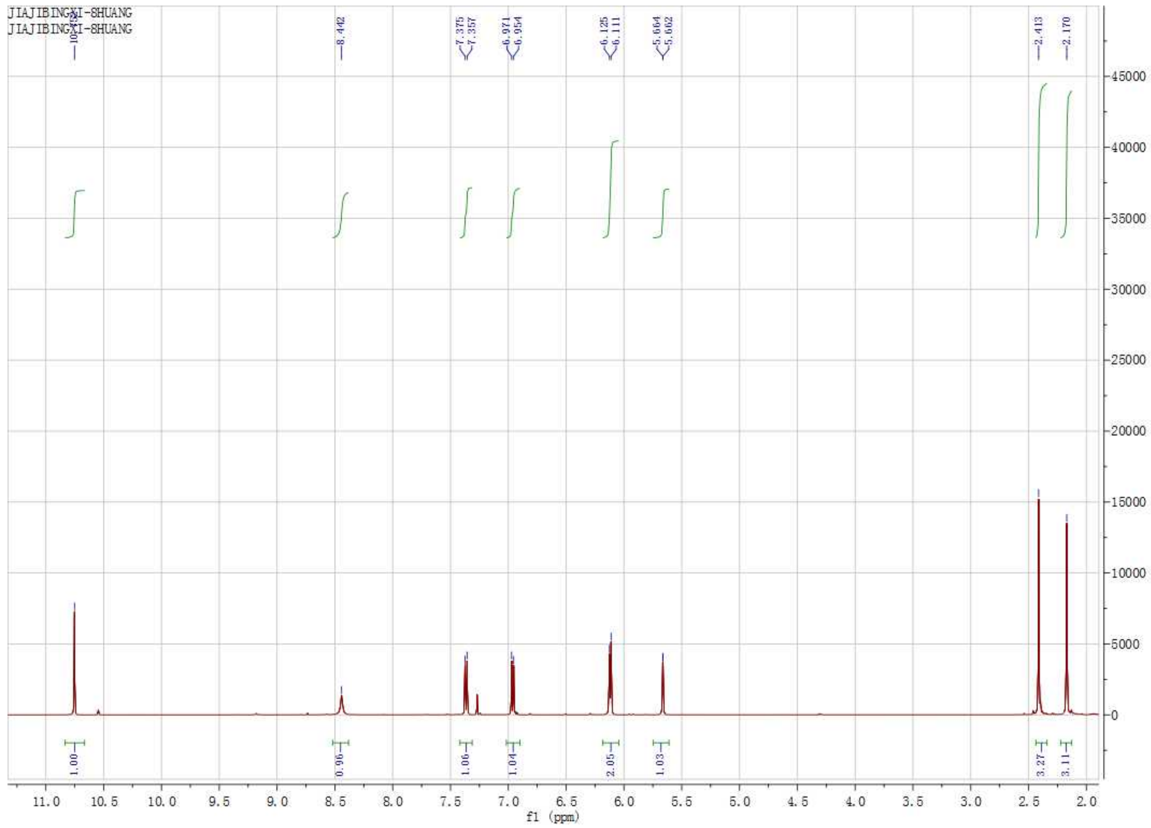

Figure S15. ^{1}H NMR of **4bg**

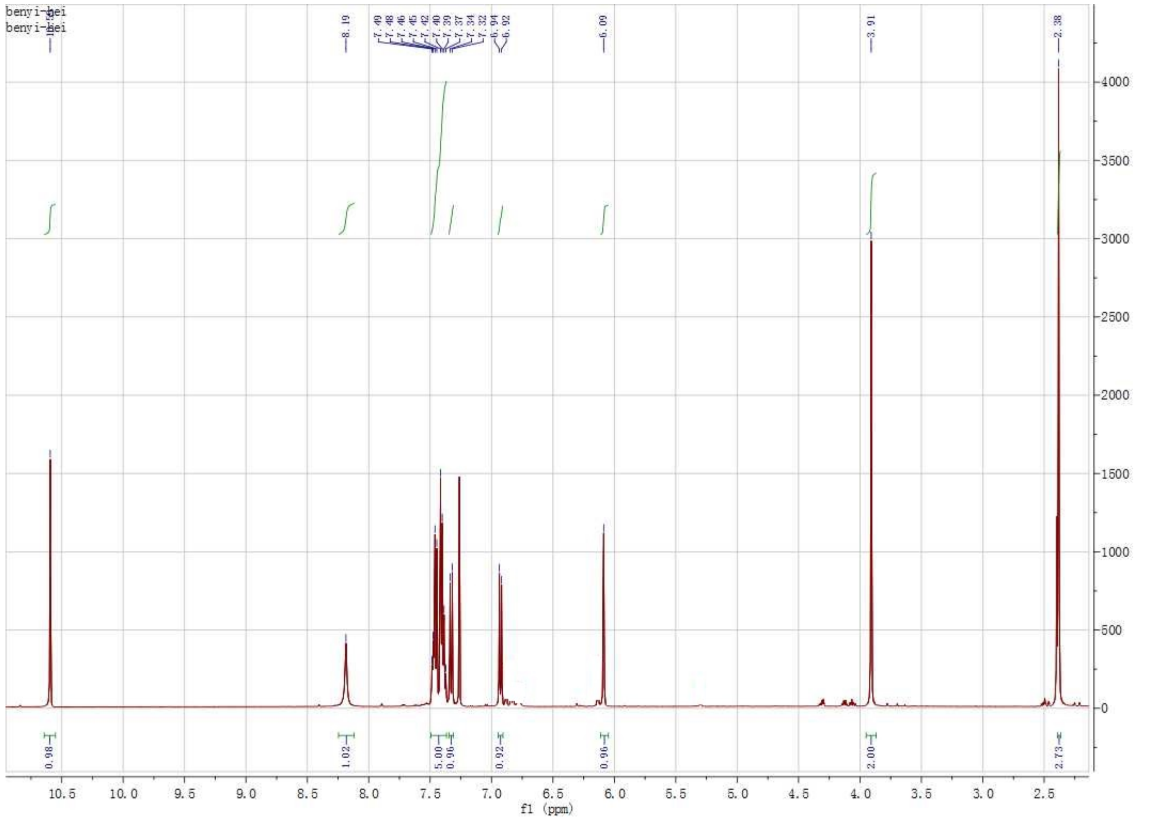

Figure S16. ^{1}H NMR of **4bh**

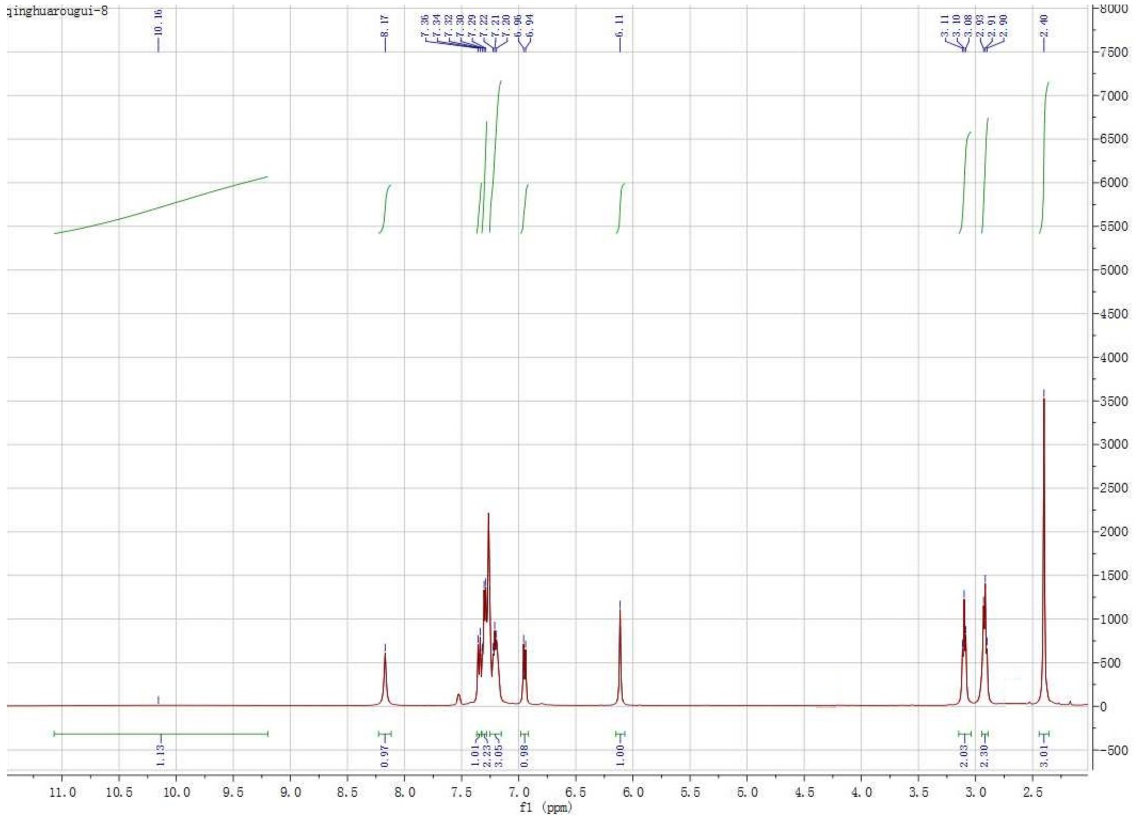

Figure S17. ^{1}H NMR of **4bi**

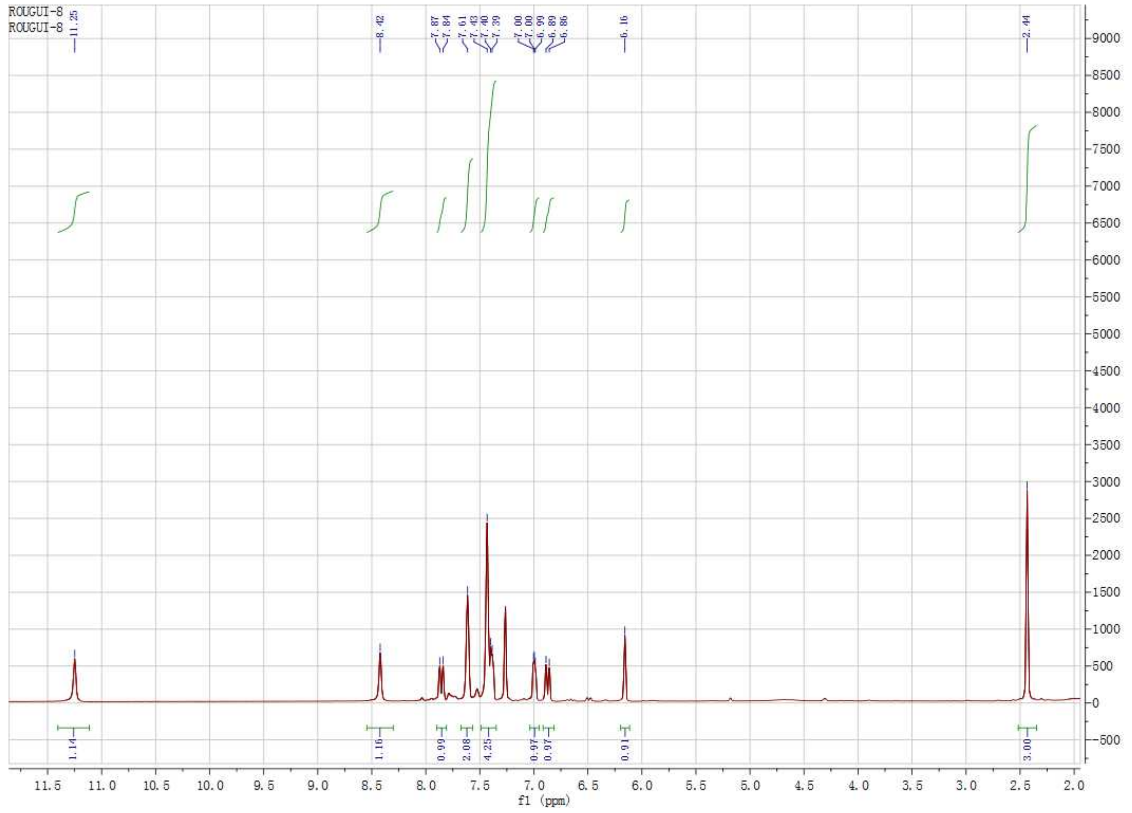

Figure S18. ^{1}H NMR of **4bj**

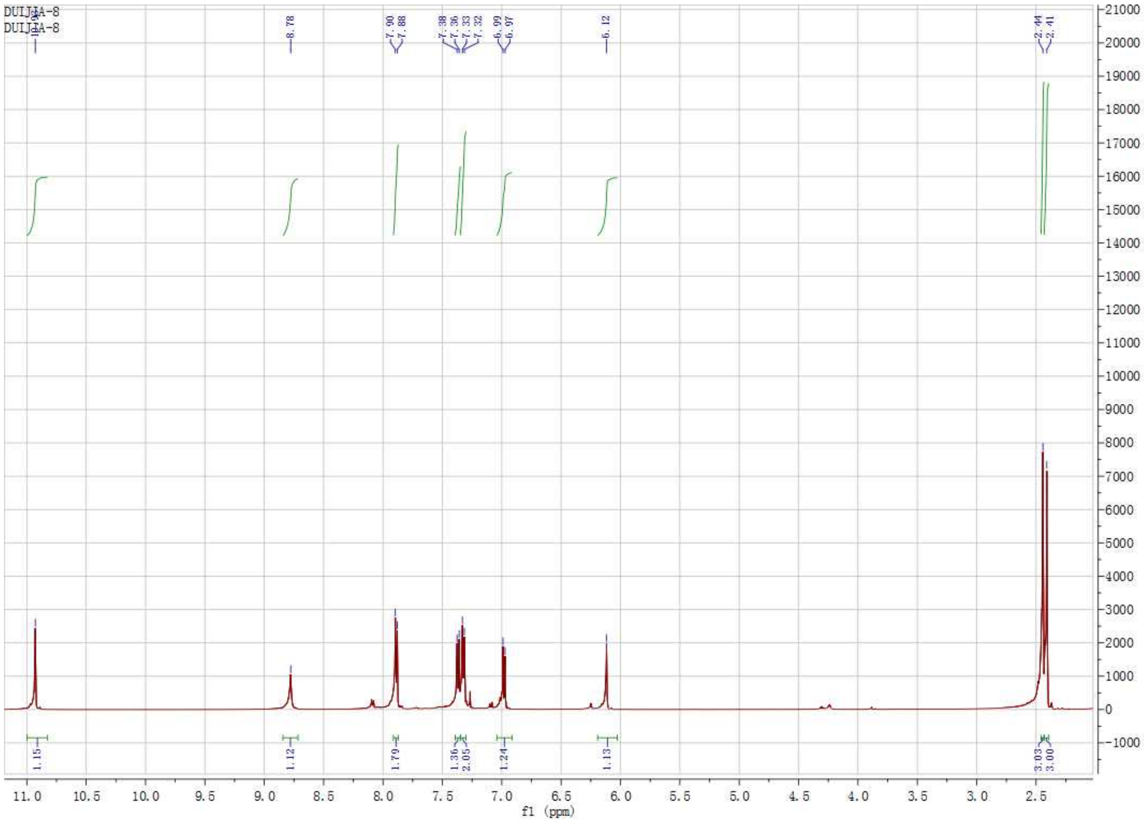

Figure S19. ^{1}H NMR of **4bk**

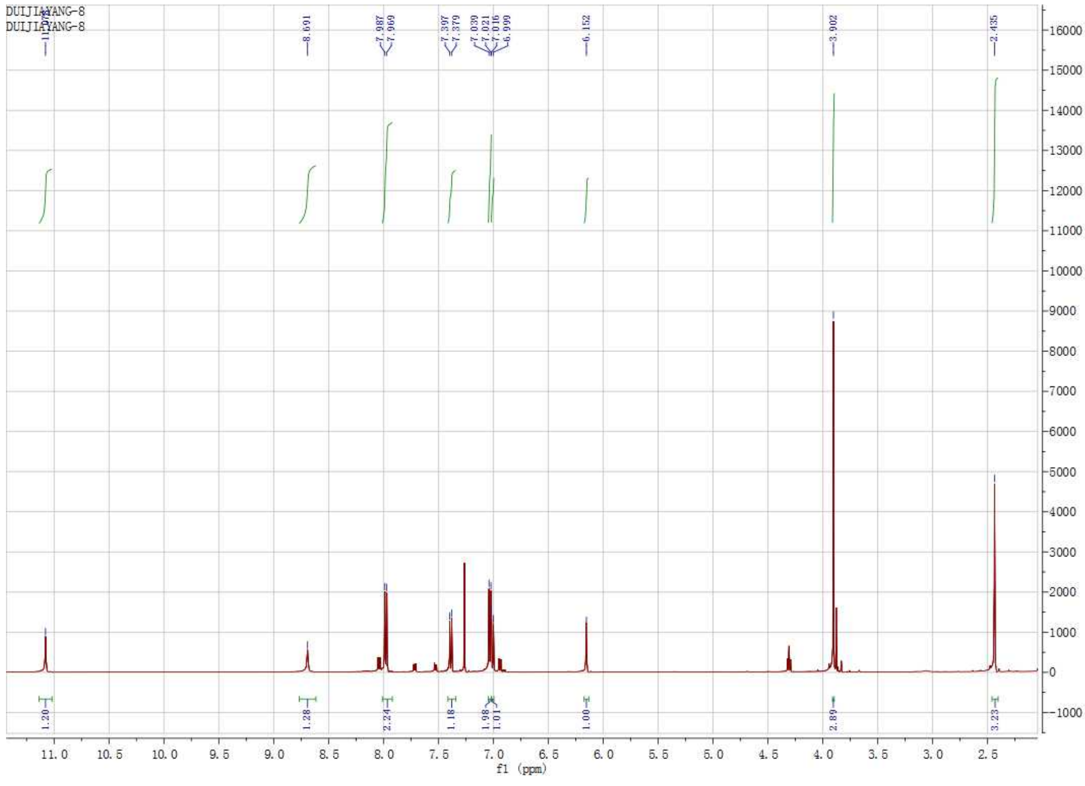

Figure S20. ^{1}H NMR of **4bl**

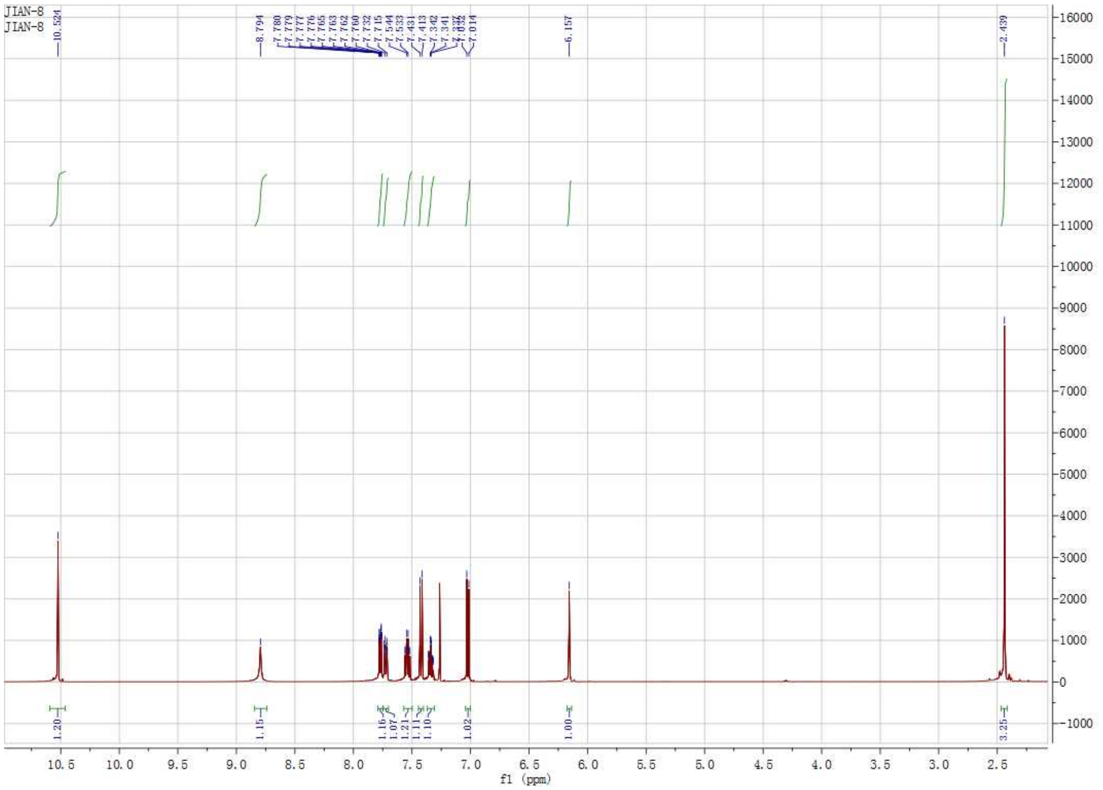

Figure S21. ^{1}H NMR of **4bm**

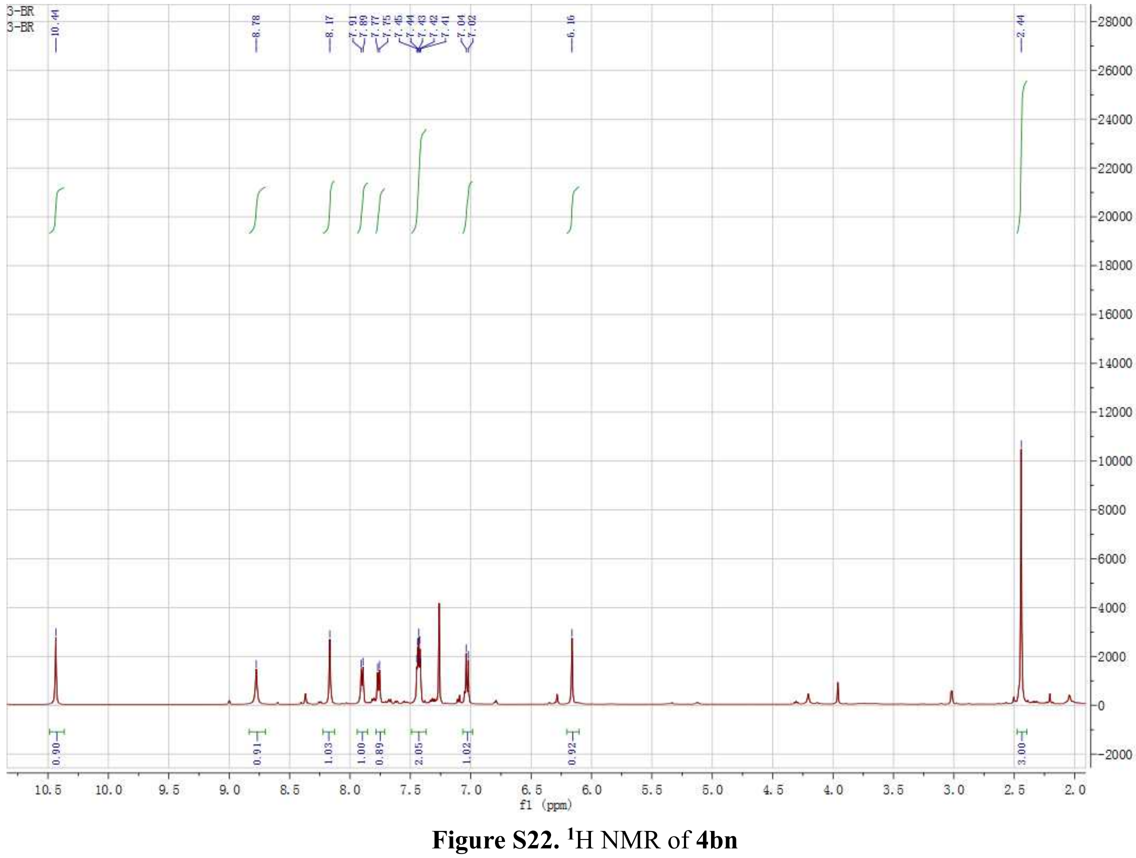

Figure S22. ^{1}H NMR of **4bn**

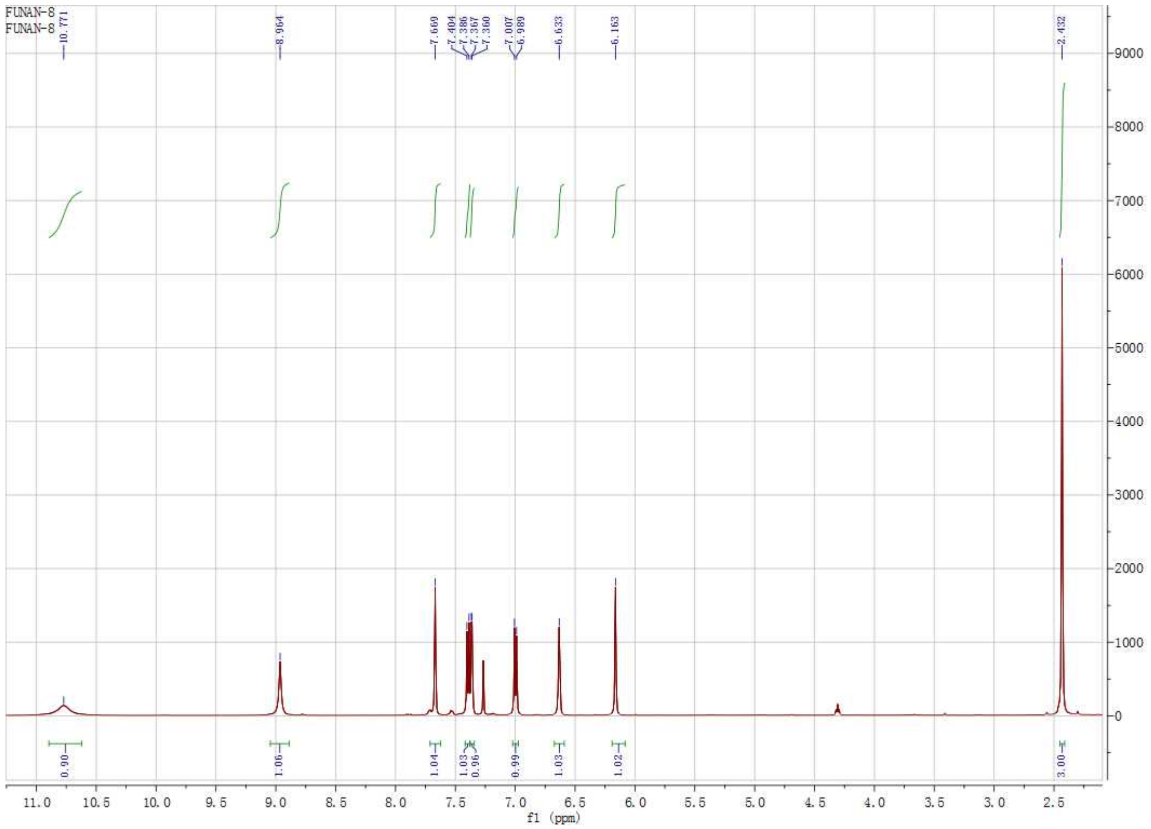

Figure S23. ^{1}H NMR of **4bo**

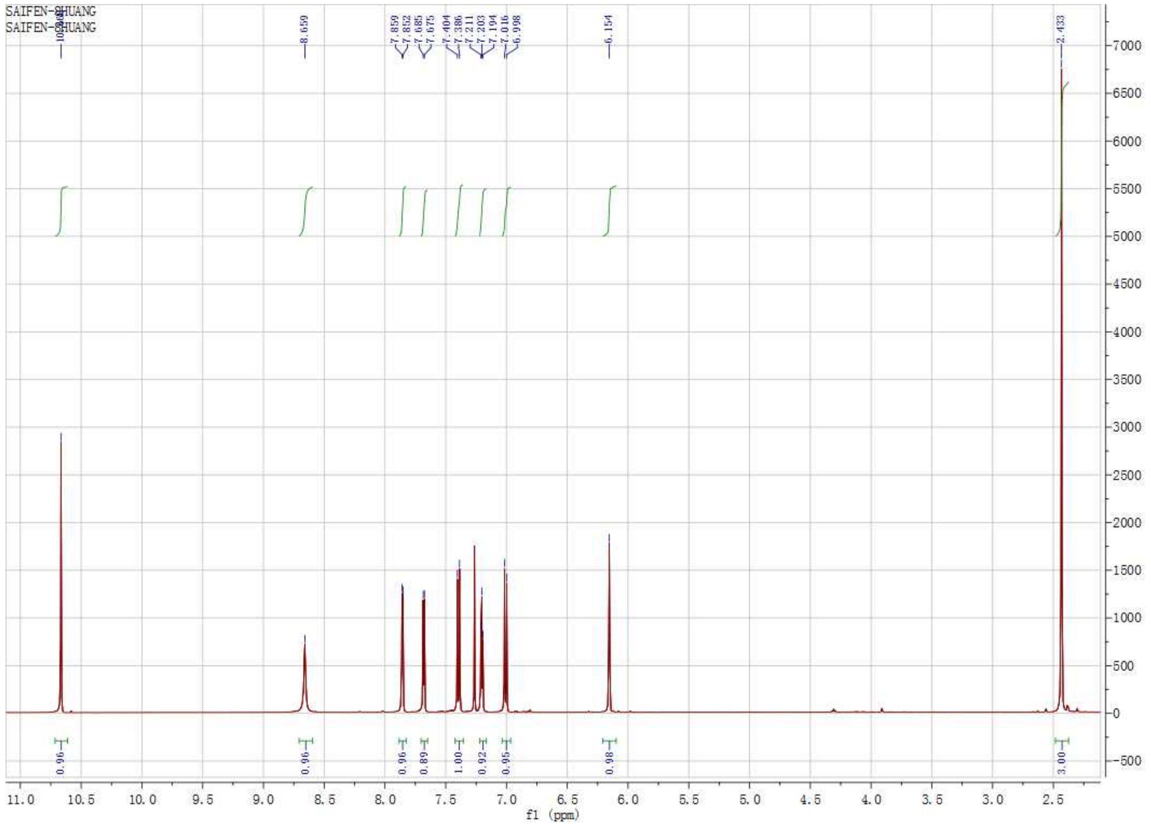

Figure S24. ^{1}H NMR of **4bp**

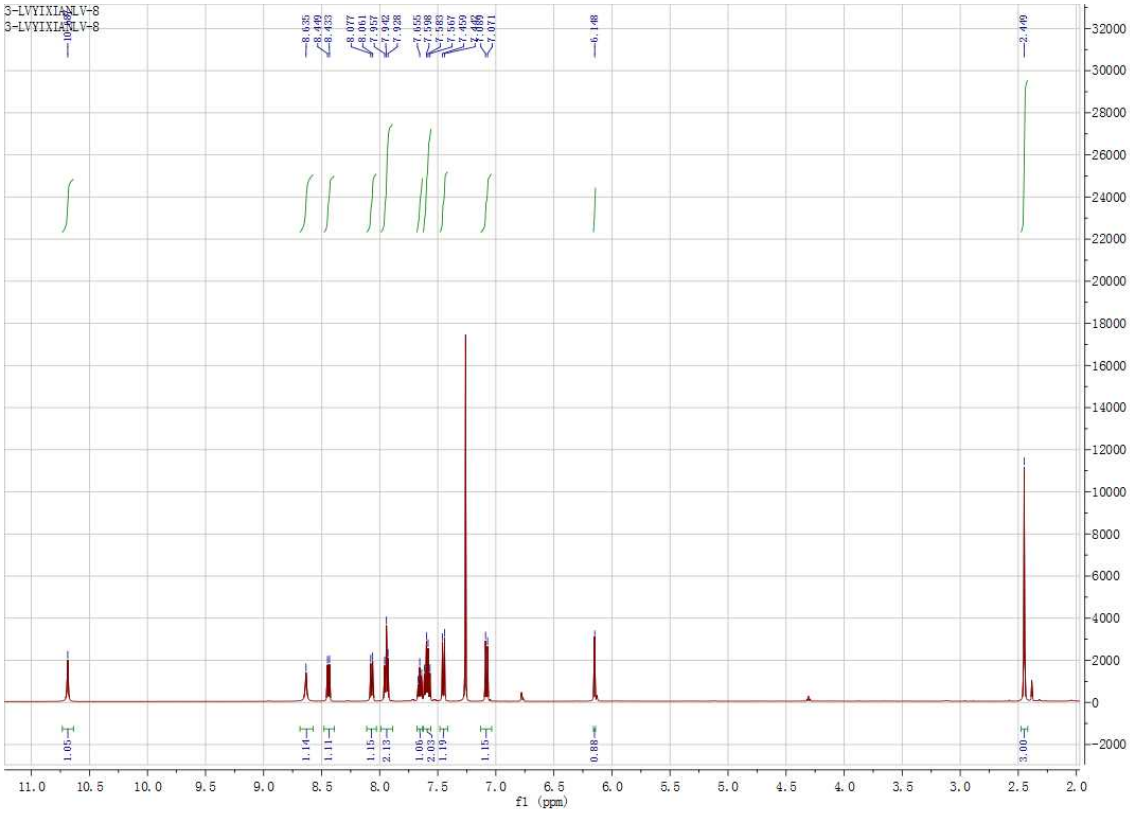

Figure S25. ^{1}H NMR of **4bq**

The ^{13}C NMR Spectra of Compounds **4aa-4bq**.

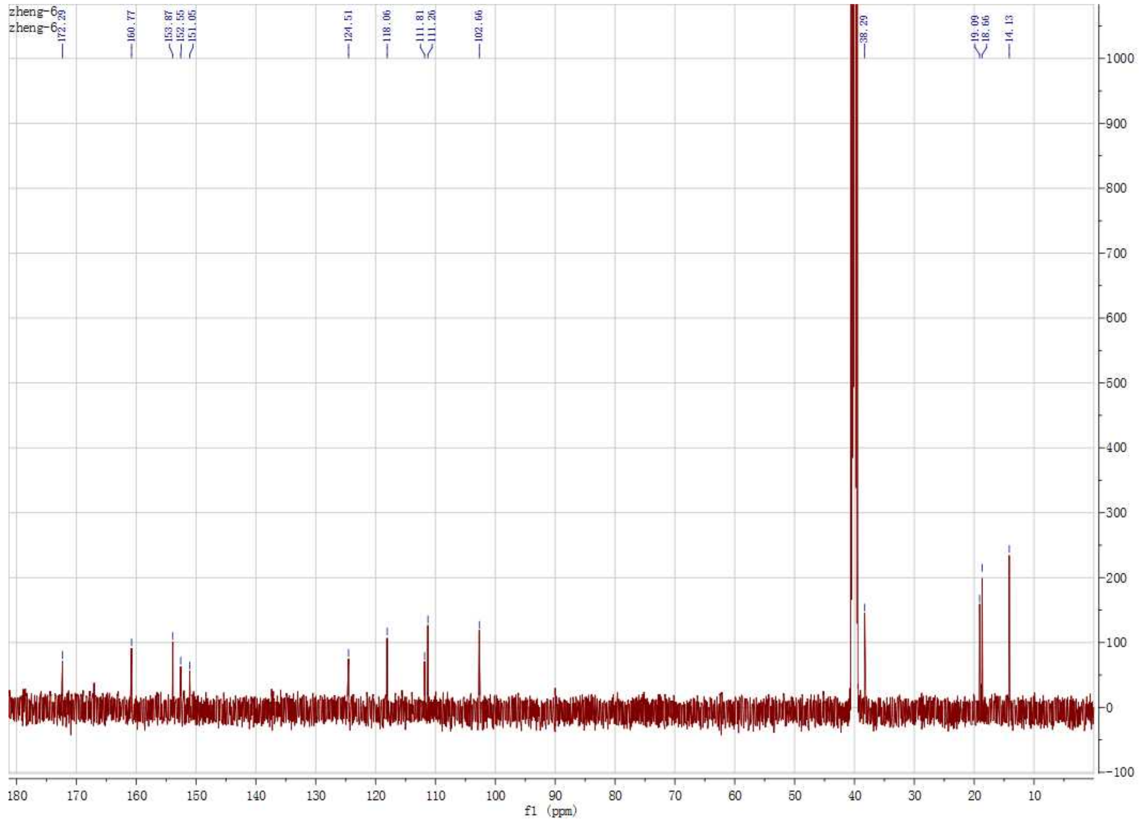

Figure S26. ^{13}C NMR of **4aa**

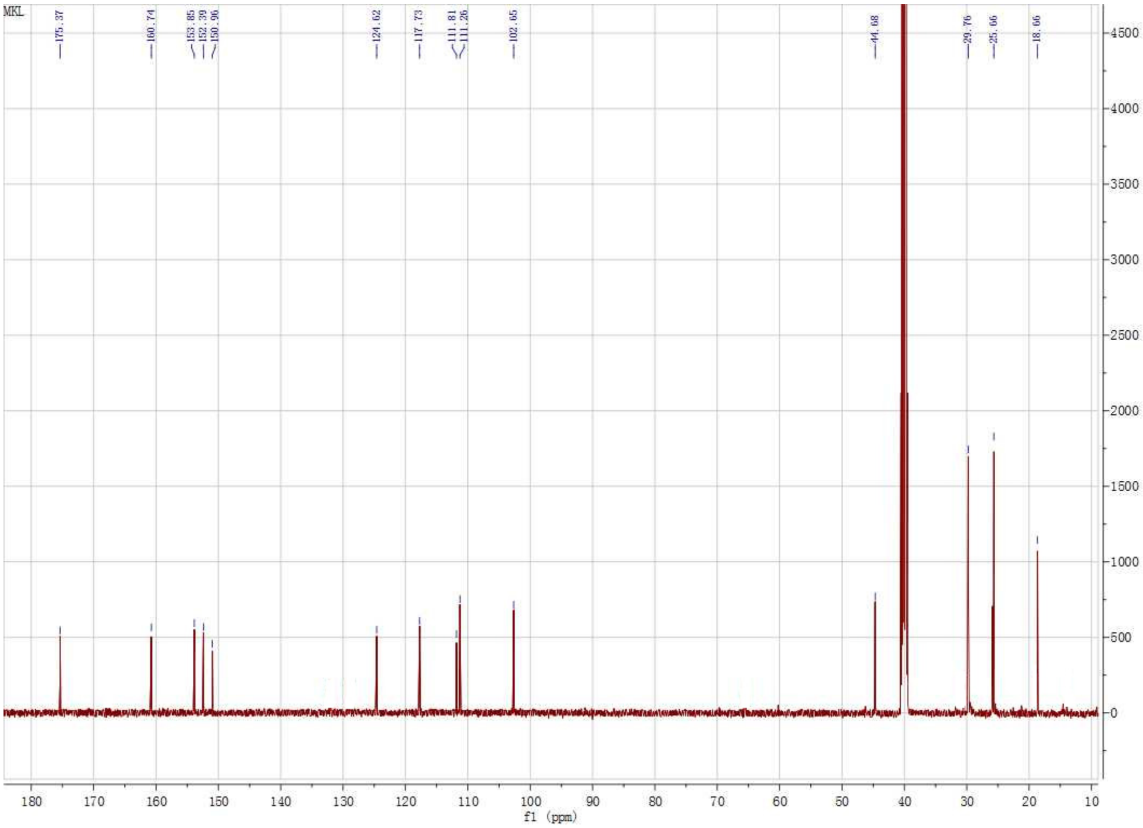

Figure S27. ^{13}C NMR of **4ab**

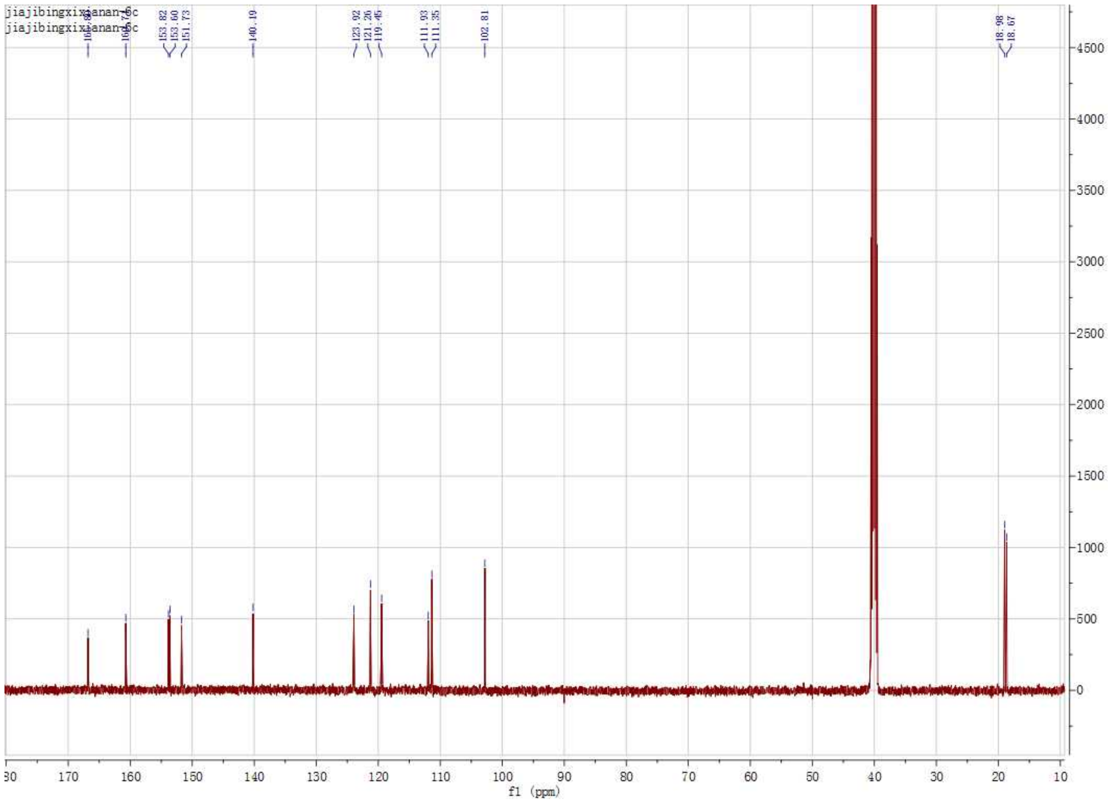

Figure S28. ^{13}C NMR of **4ac**

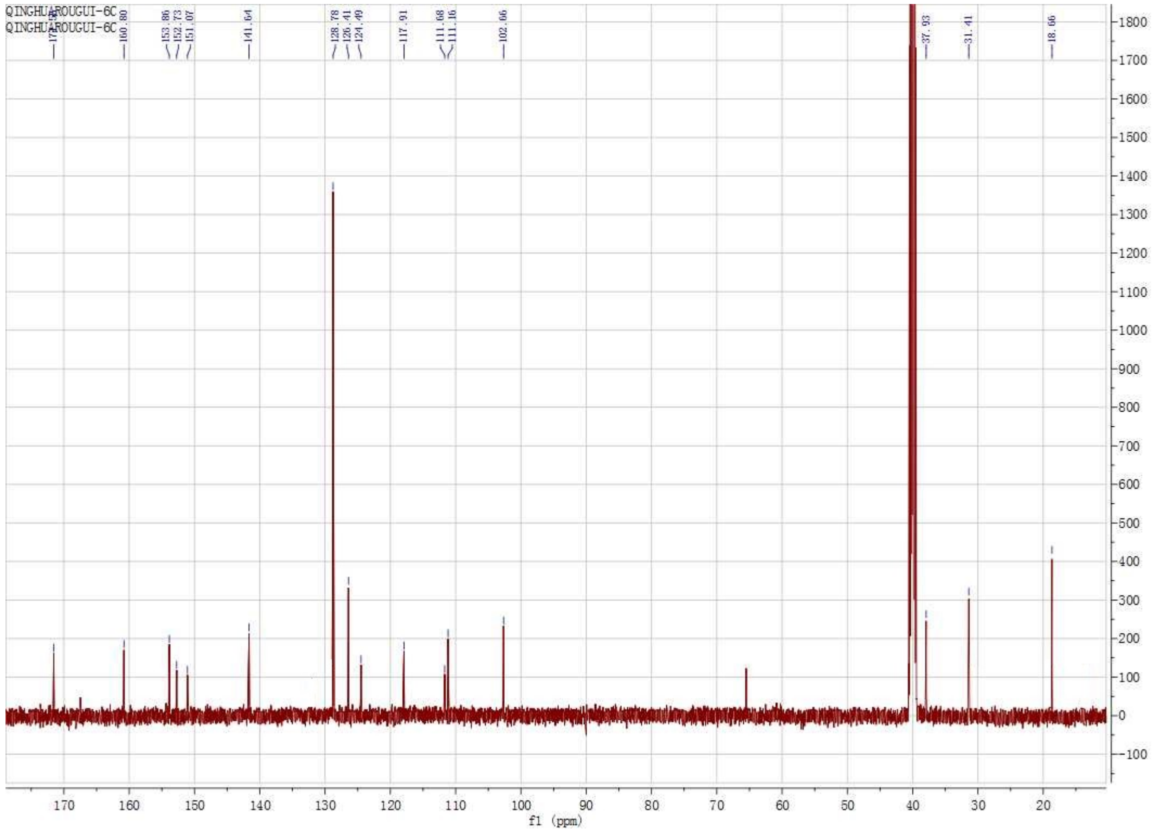

Figure S29. ^{13}C NMR of **4ad**

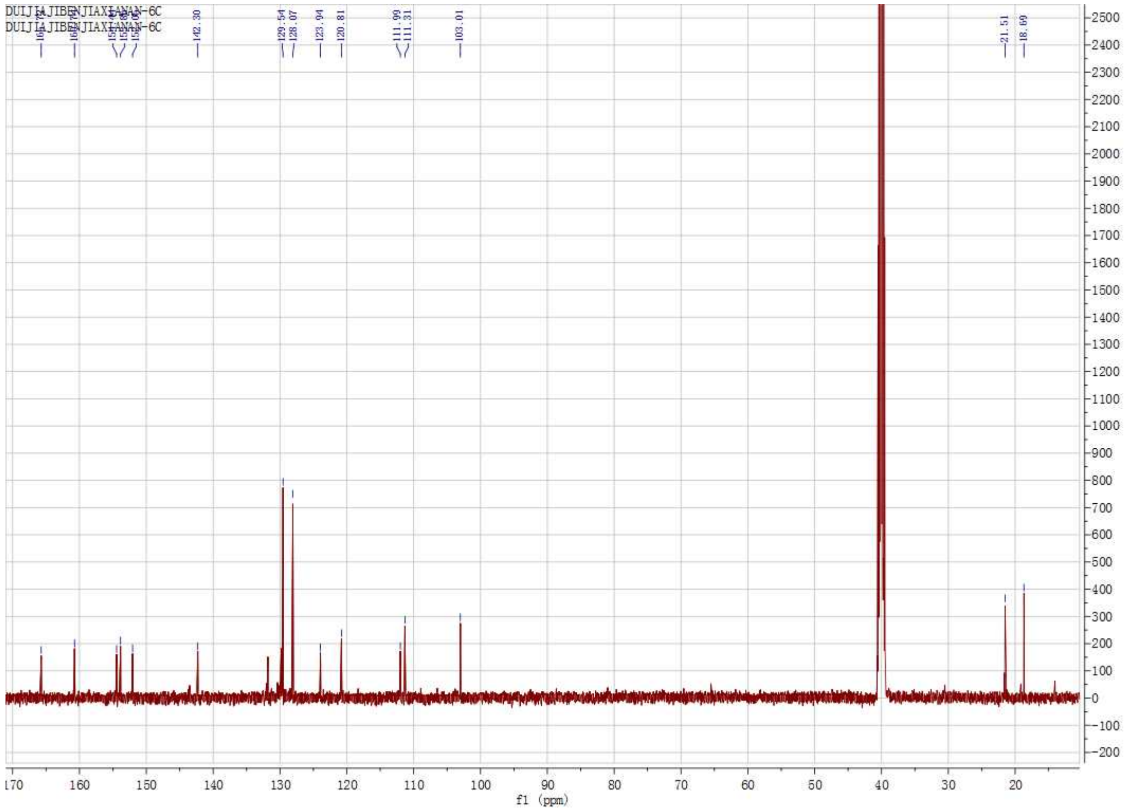

Figure S30. ^{13}C NMR of **4ae**

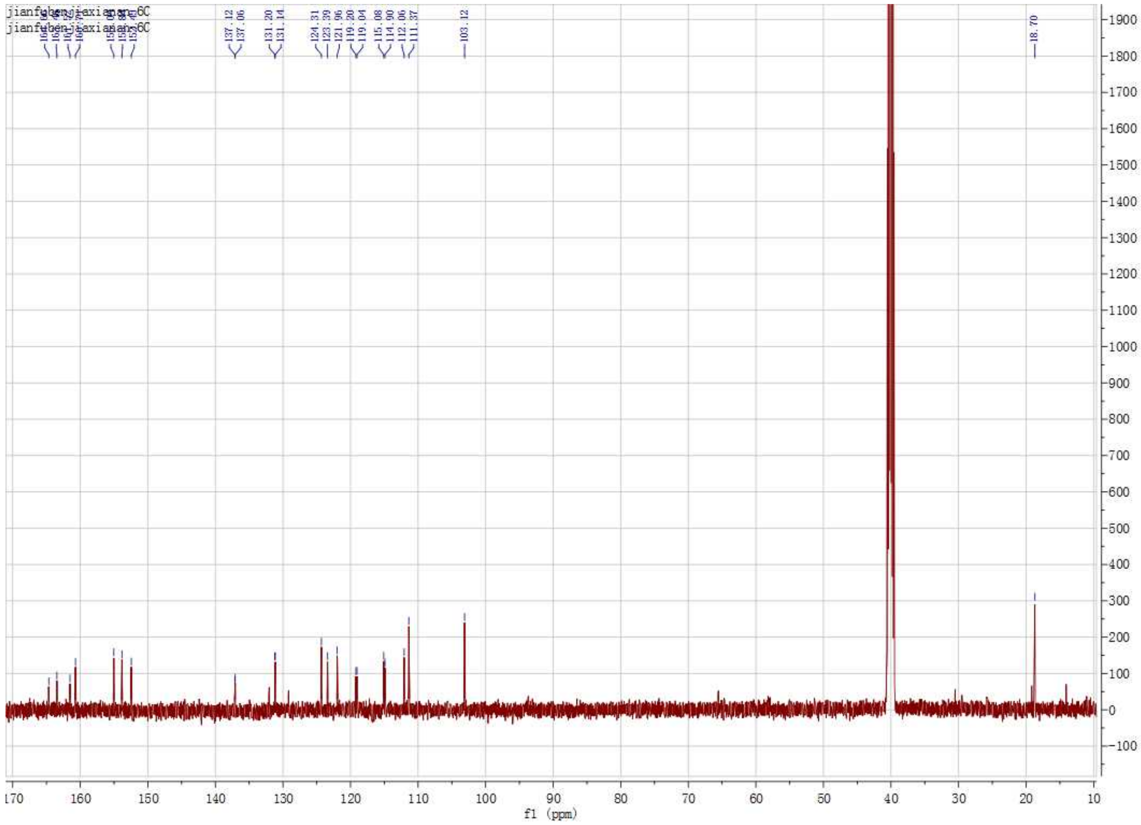

Figure S31. ^{13}C NMR of **4af**

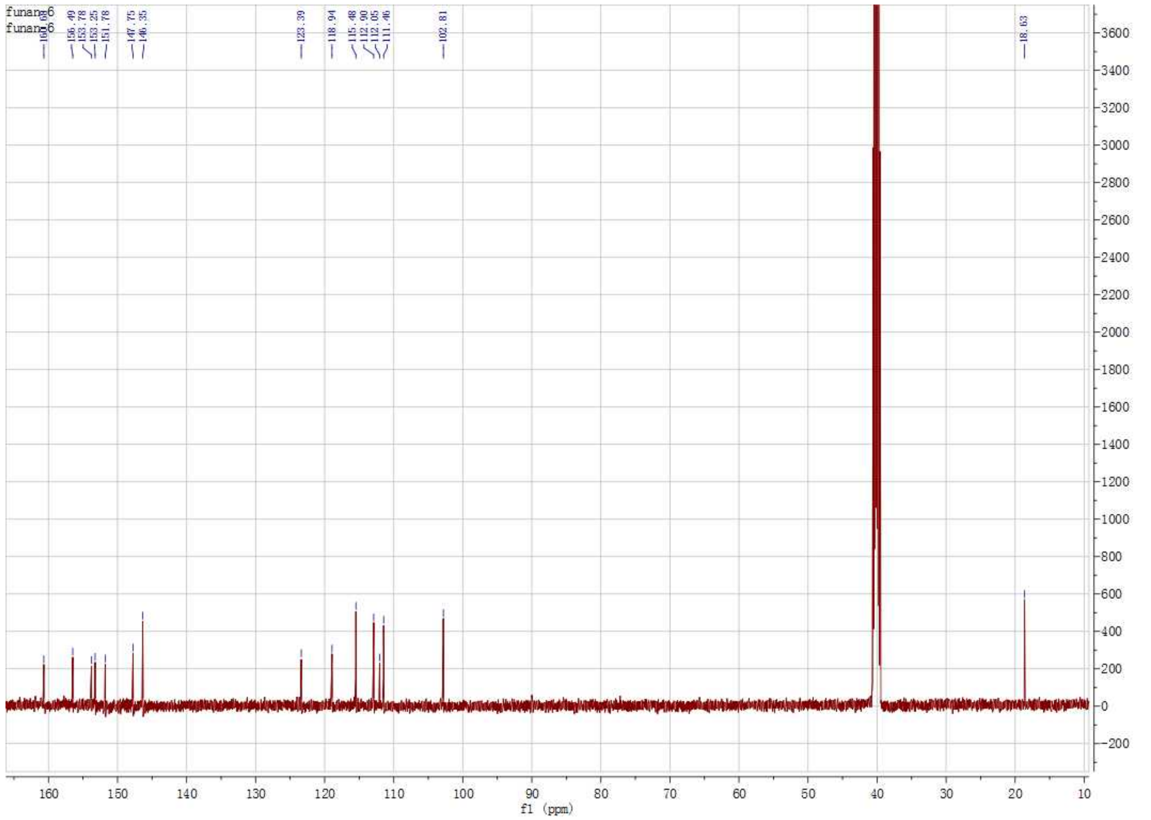

Figure S32. ^{13}C NMR of **4ag**

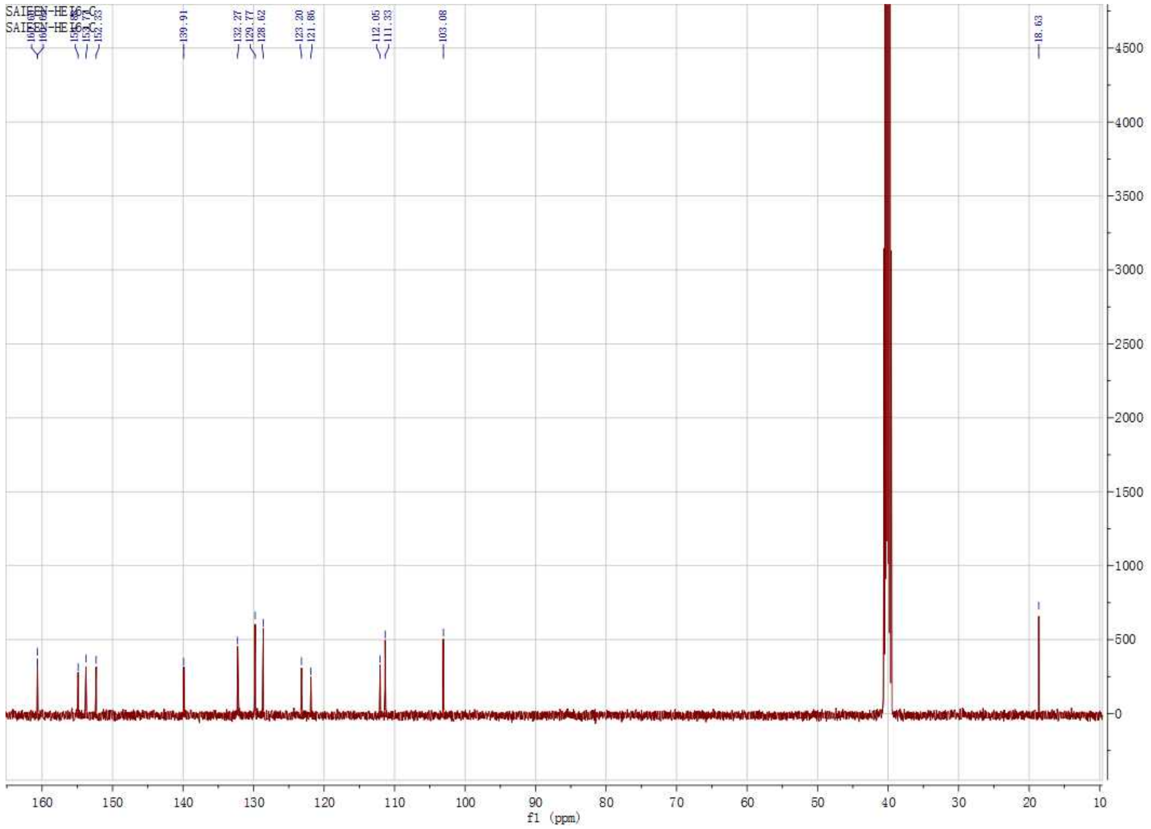

Figure S33. ^{13}C NMR of **4ah**

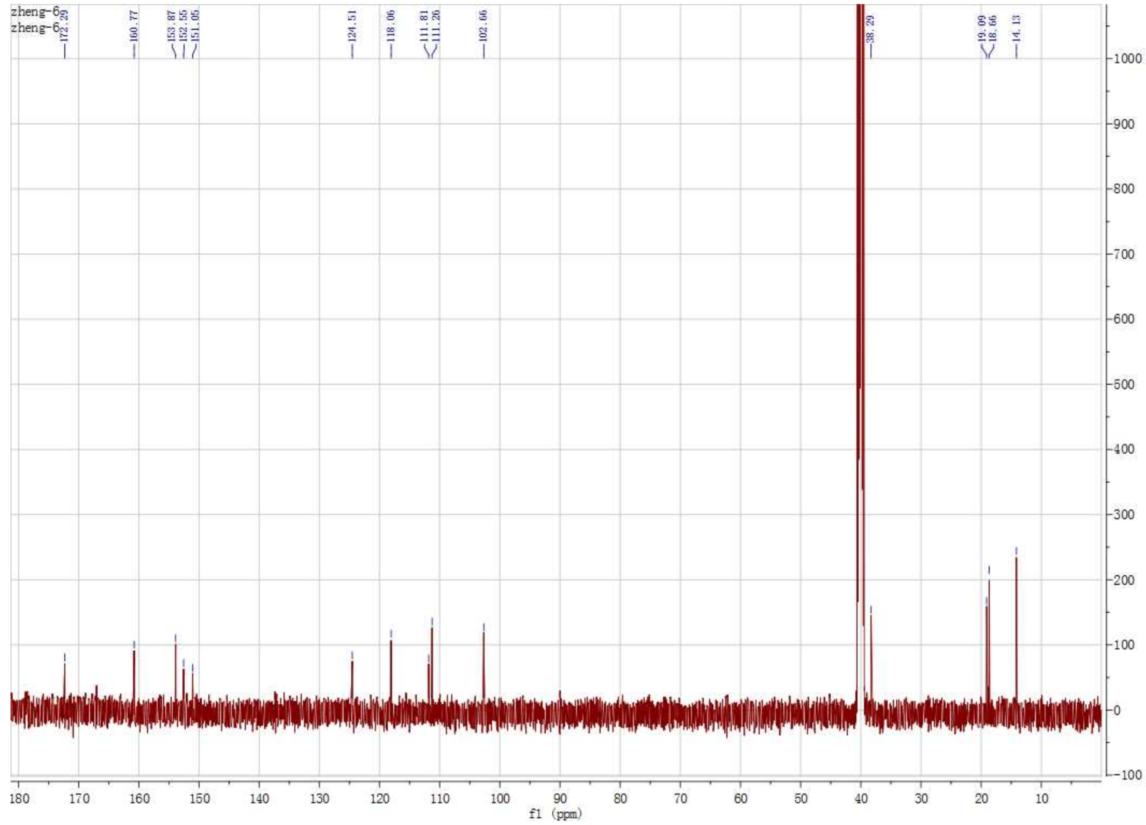

Figure S34. ^{13}C NMR of **4ba**

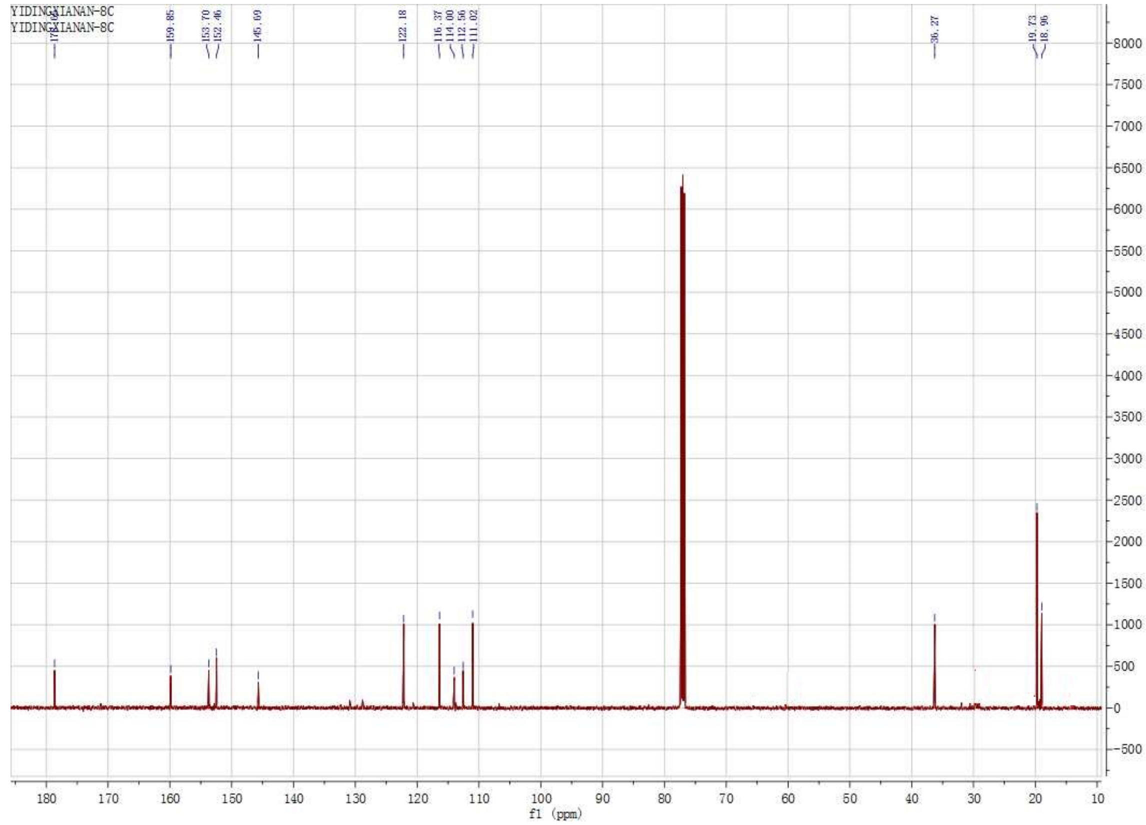

Figure S35. ^{13}C NMR of **4bb**

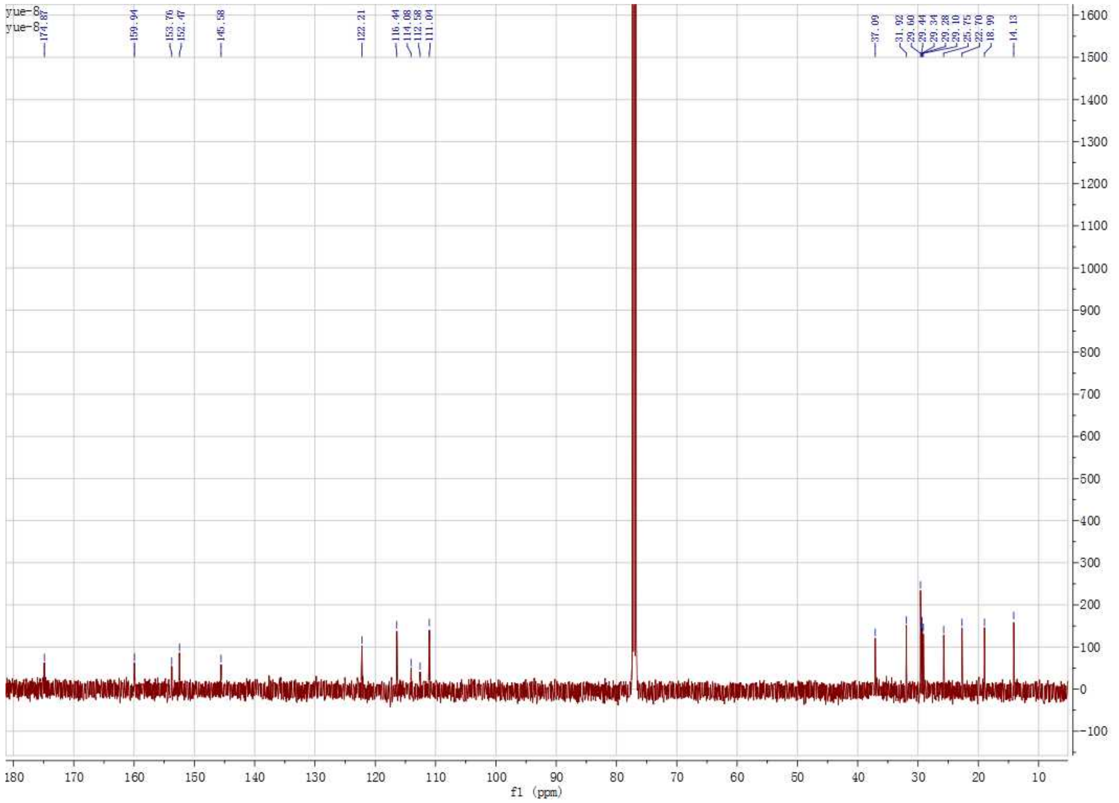

Figure S36. ^{13}C NMR of **4bc**

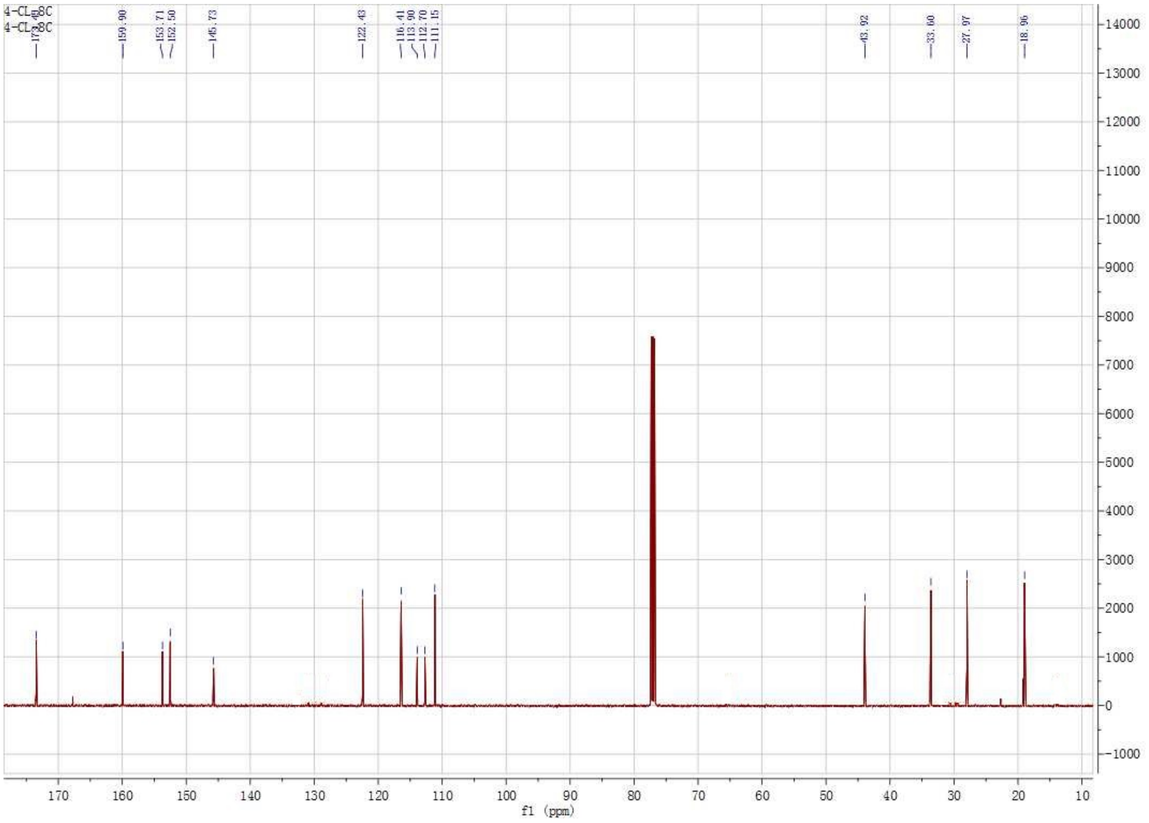

Figure S37. ^{13}C NMR of **4bd**

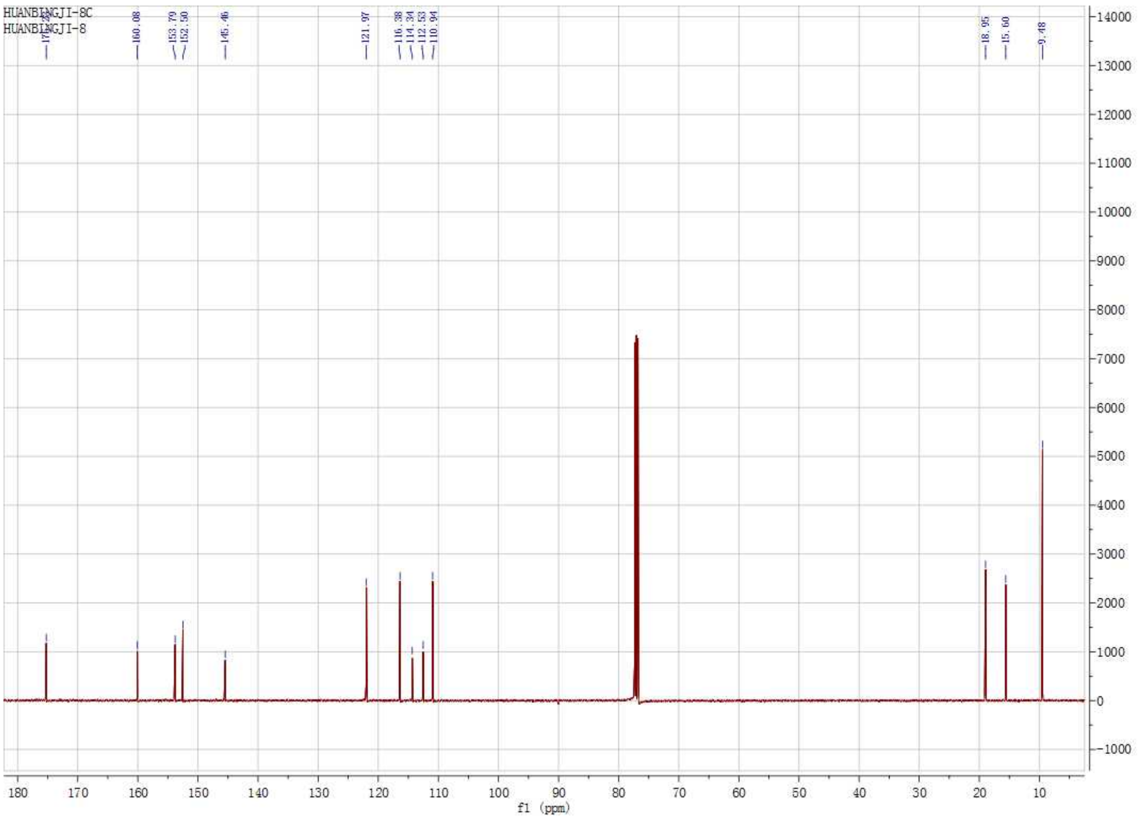

Figure S38. ^{13}C NMR of **4be**

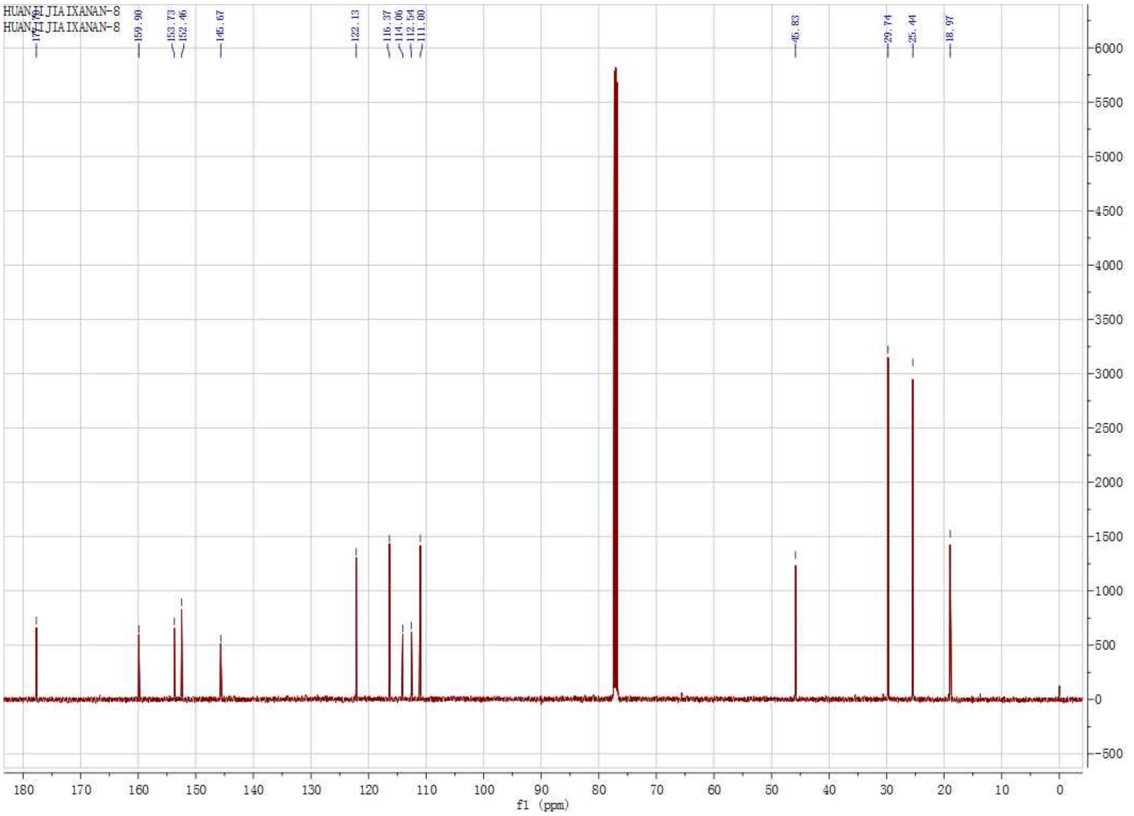

Figure S39. ^{13}C NMR of **4bf**

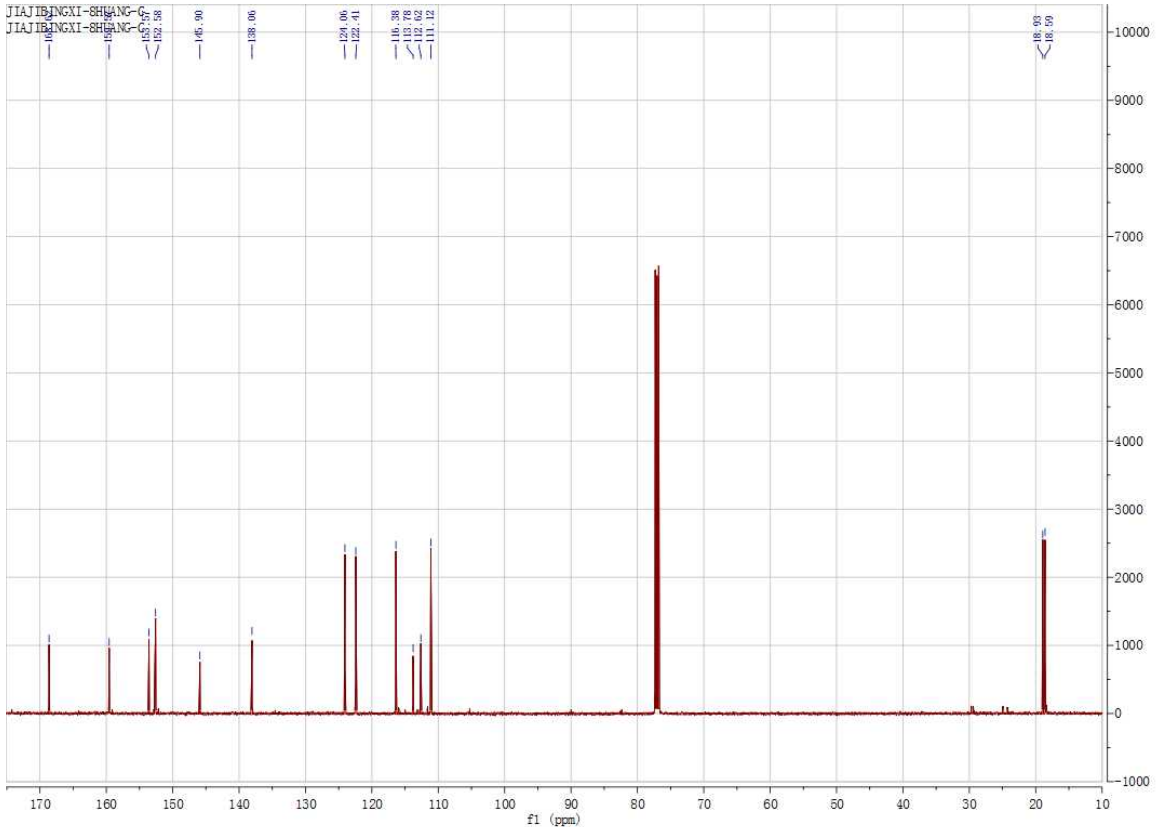

Figure S40. ^{13}C NMR of **4bg**

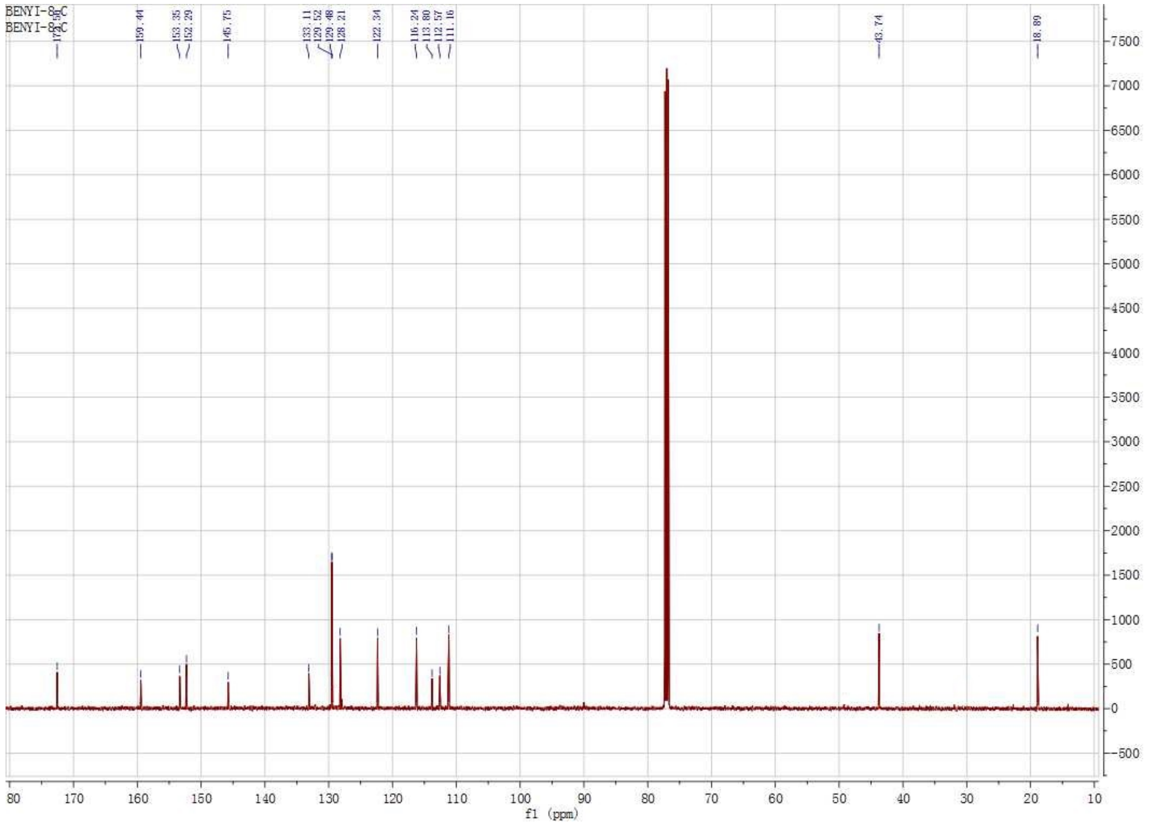

Figure S41. ^{13}C NMR of **4bh**

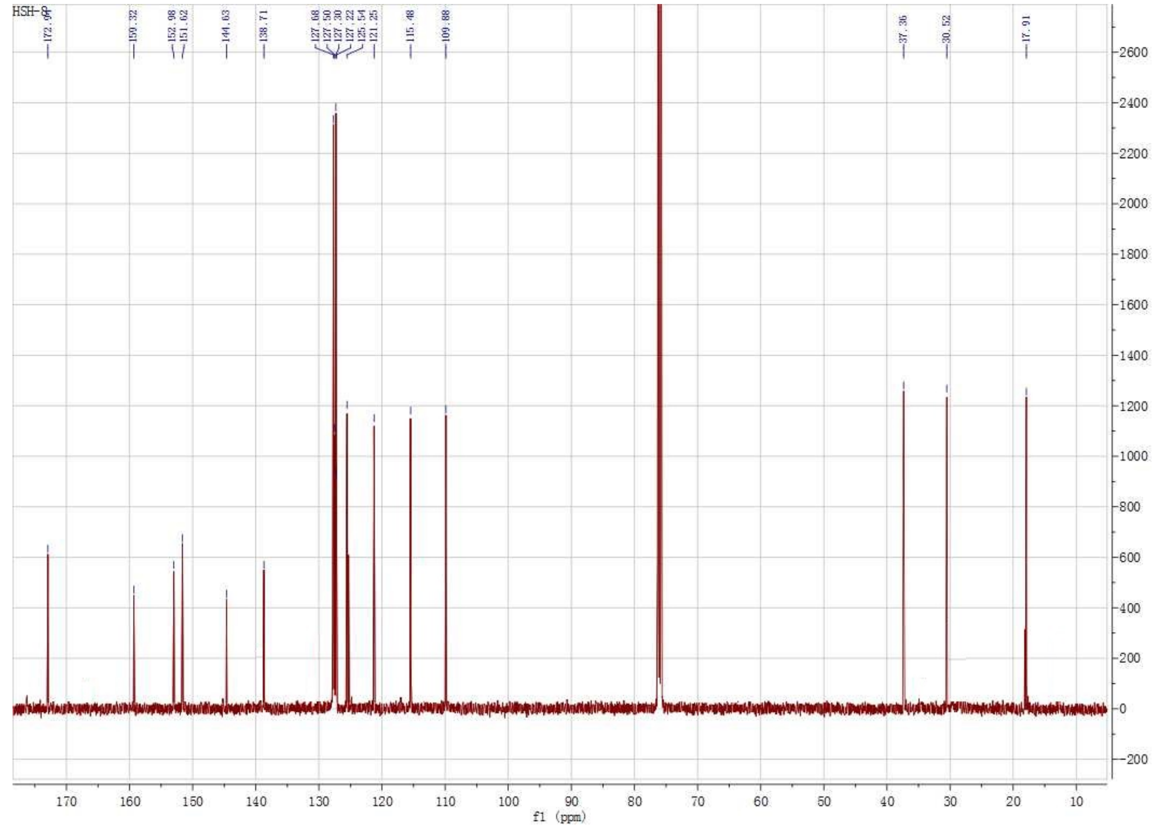

Figure S42. ^{13}C NMR of **4bi**

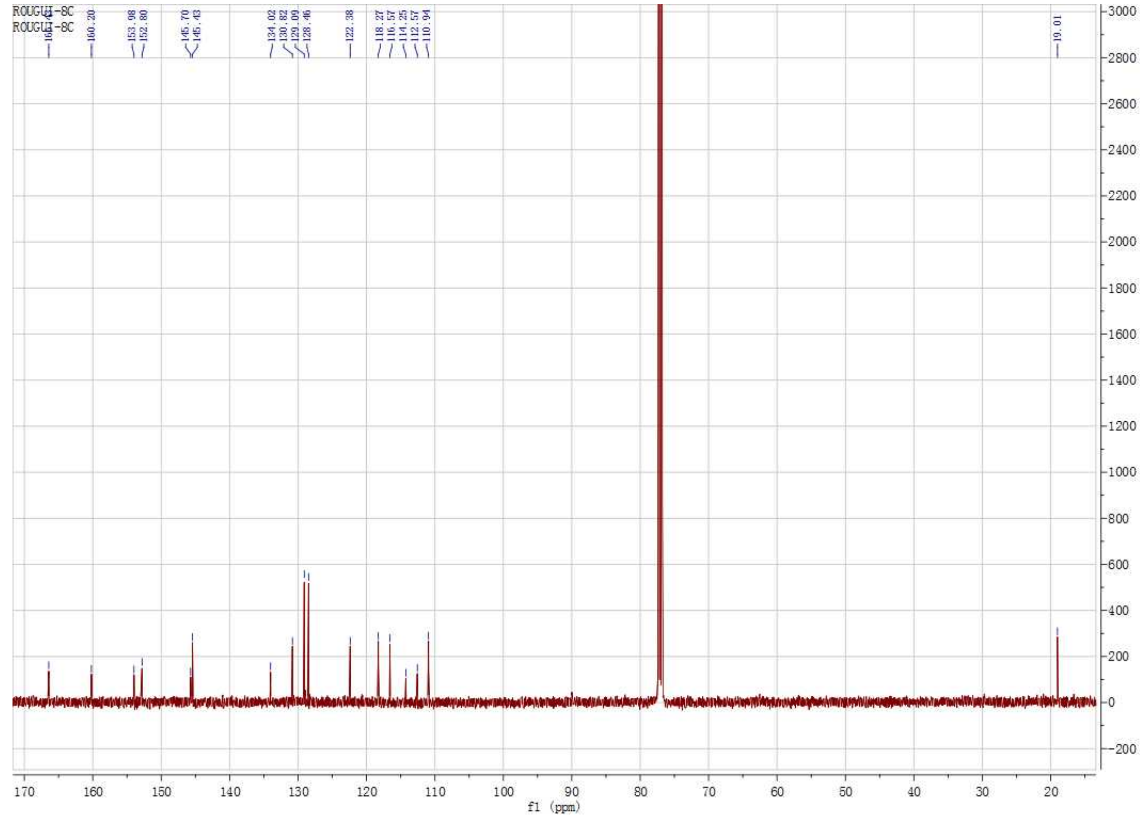

Figure S43. ^{13}C NMR of **4bj**

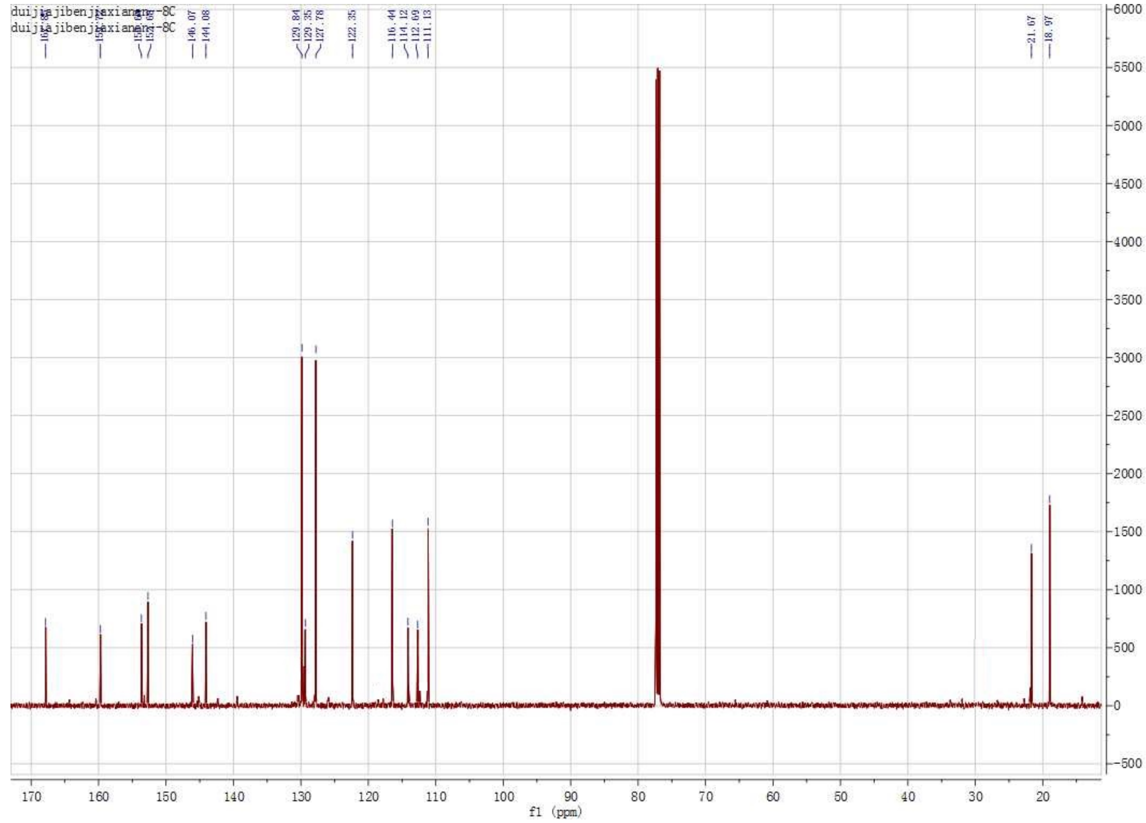

Figure S44. ^{13}C NMR of **4bk**

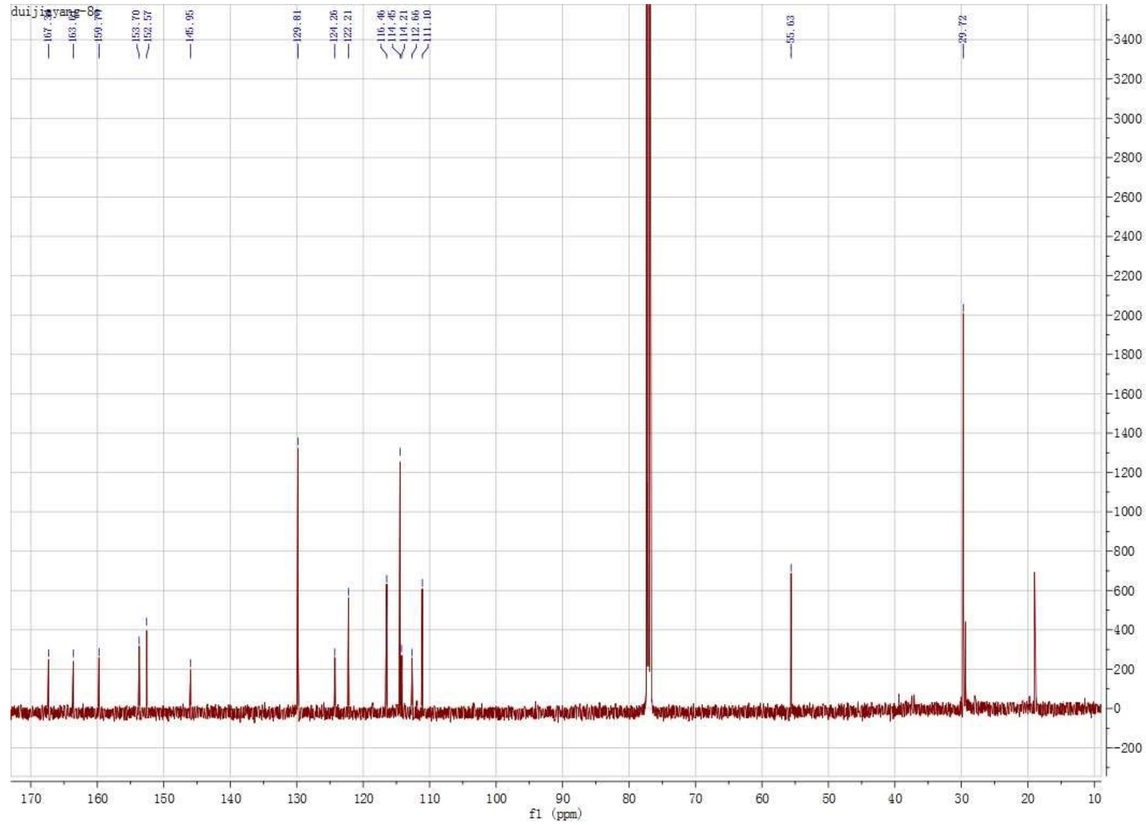

Figure S45. ^{13}C NMR of **4bl**

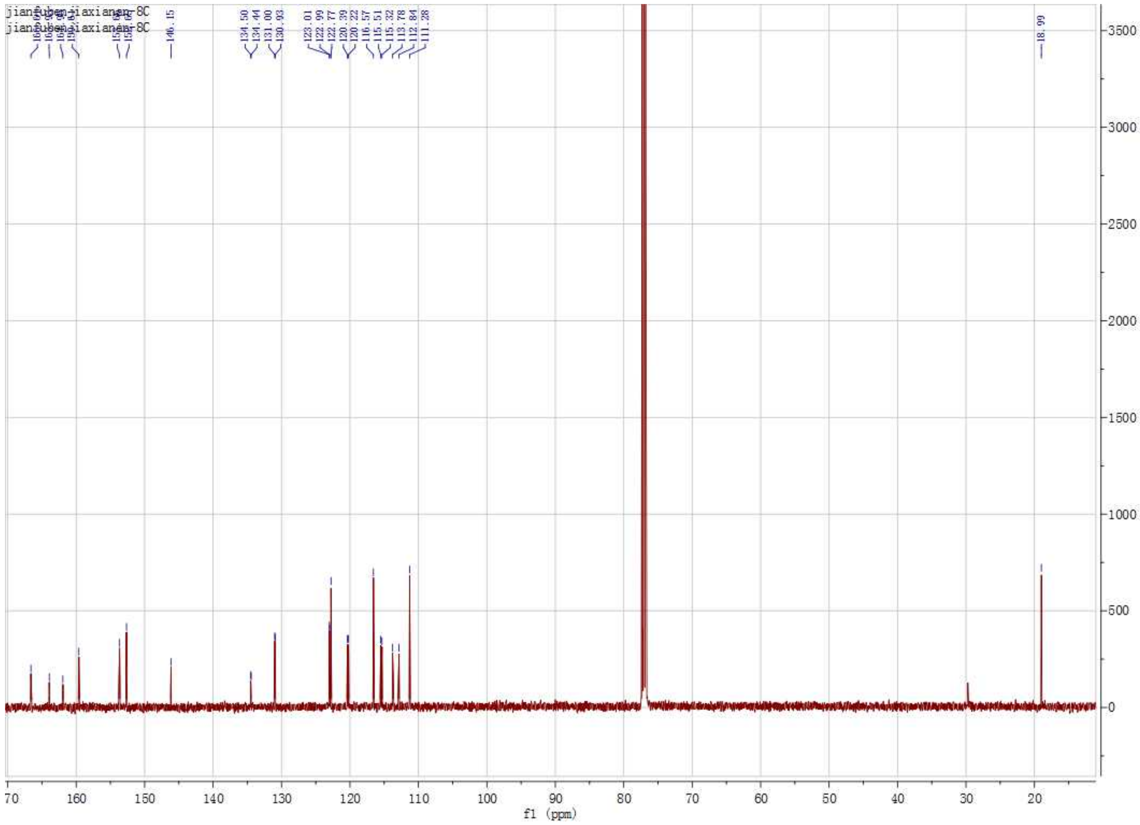

Figure S46. ^{13}C NMR of **4bm**

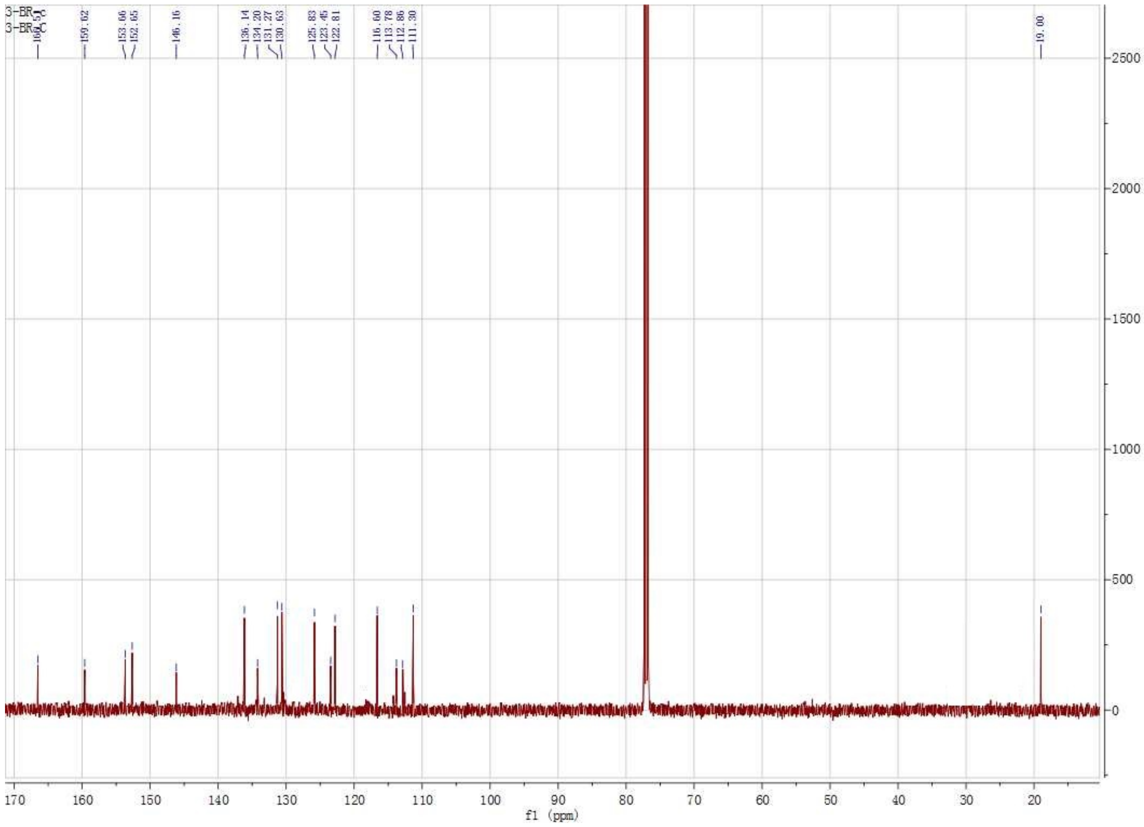

Figure S47. ^{13}C NMR of **4bn**

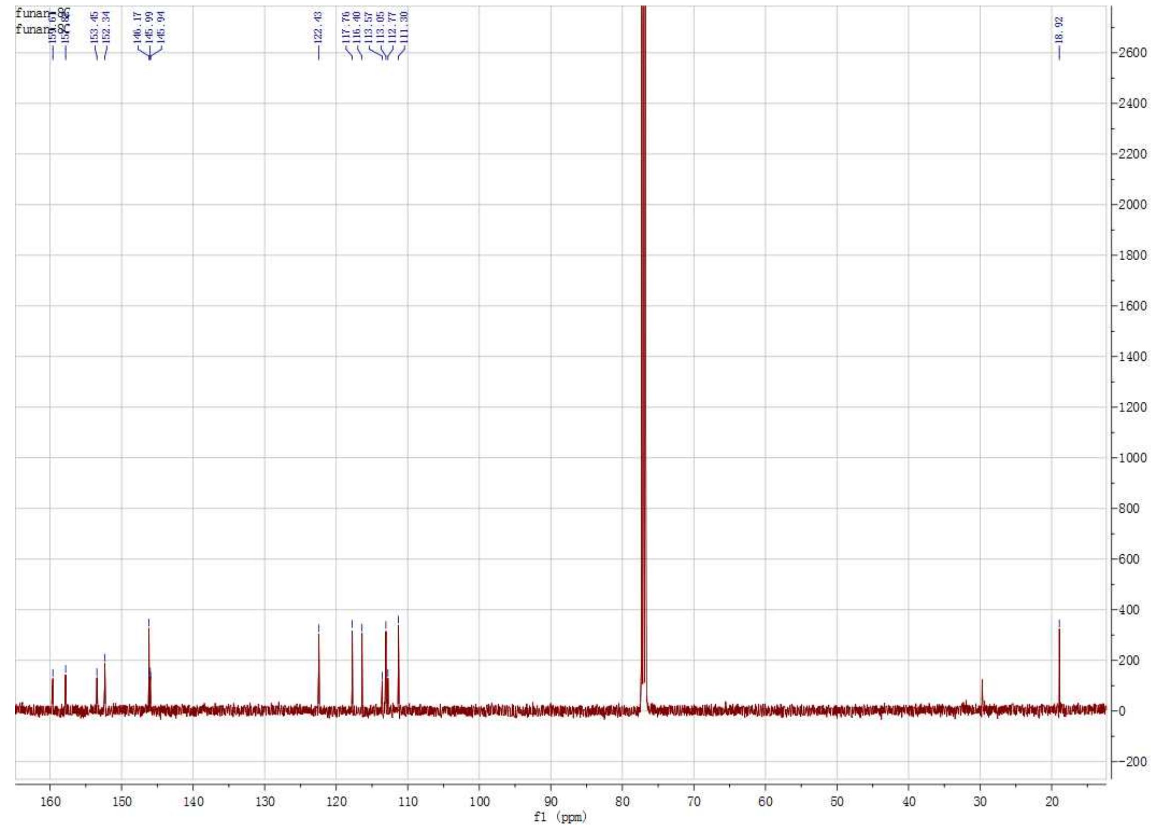

Figure S48. ^{13}C NMR of **4bo**

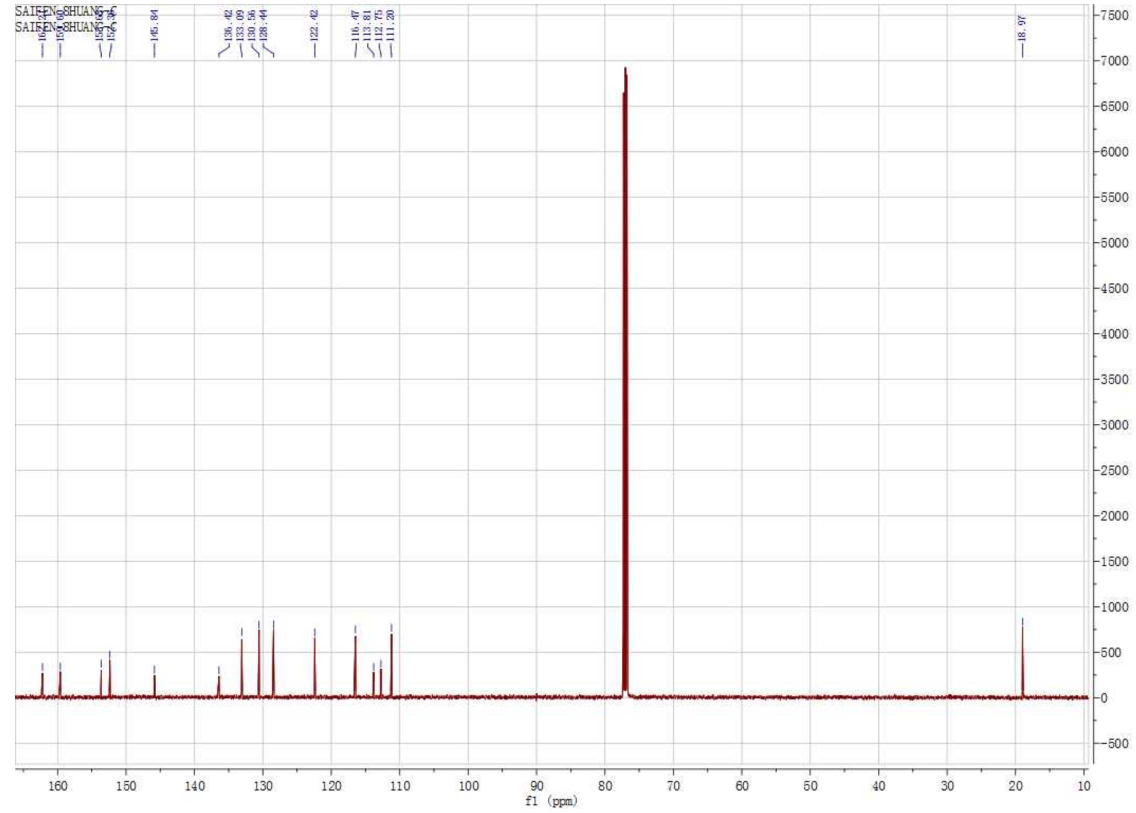

Figure S49. ^{13}C NMR of **4bp**

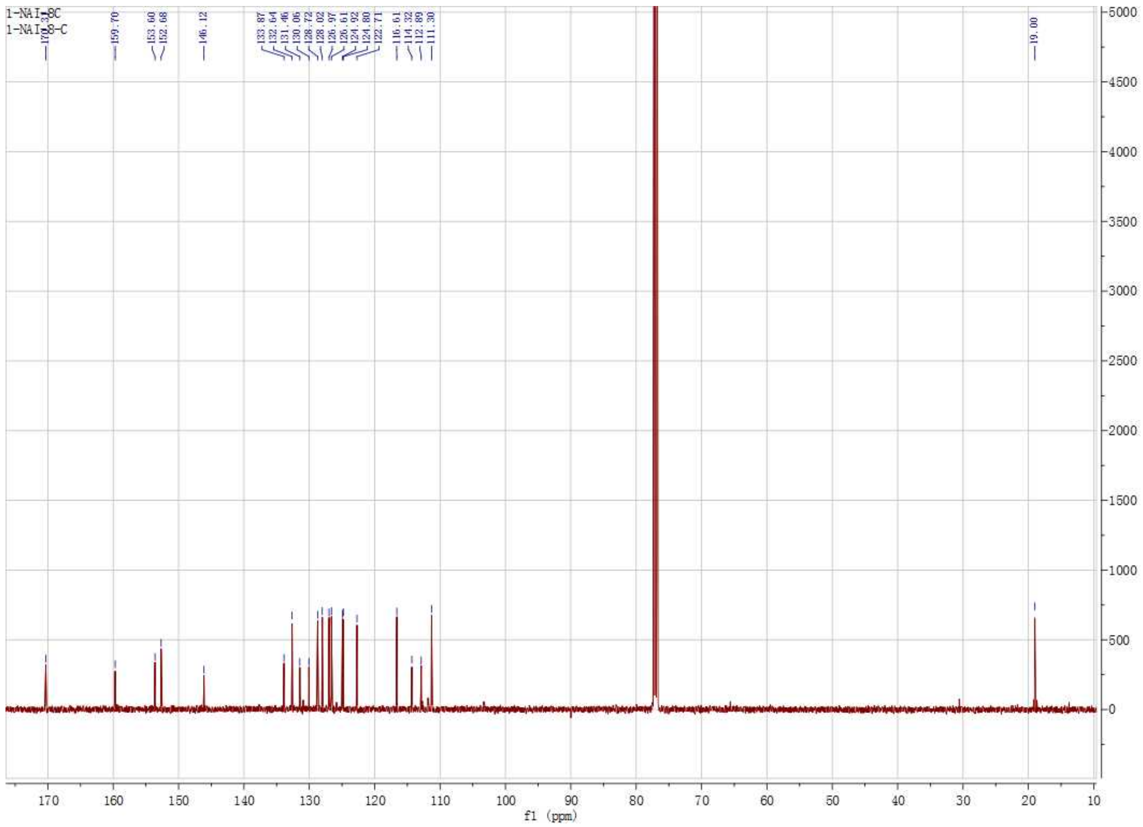

Figure S50. ^{13}C NMR of **4bq**
